# Multimodal characterization of temperature-induced structural remodeling and aggregation of regenerated silk fibroin

**DOI:** 10.1039/d6ra06368c

**Published:** 2026-10-02

**Authors:** Shaik Basha, Darshan Chikkanayakanahalli Mukunda, Ameera K, Ahana Chattopadhyay, Krishna Kishore Mahato

**Affiliations:** a Department of Biophysics, Manipal School of Life Sciences, Manipal Academy of Higher Education Manipal India mahato.kk@manipal.edu kkmahato@gmail.com; b Department of Biosciences & Bioengineering, Indian Institute of Technology Dharwad Dharwad-580011 Karnataka India

## Abstract

Regenerated silk fibroin (RSF) is a protein biomaterial whose molecular conformation, aggregation behavior, and functional properties are strongly influenced by processing conditions. The current study investigated the structural response of RSF to thermal treatment following incubation at 50–100 °C for 3 h. Intrinsic fluorescence increased progressively with treatment temperature without an appreciable displacement of the emission maximum, while fluorescence lifetime measurements at selected conditions indicated altered excited-state behavior of the intrinsic fluorophore population. Increased Thioflavin T and Congo Red responses were accompanied by enhanced ANS fluorescence, indicating increased accessibility of ordered dye-binding structures and hydrophobic sites. Secondary-structure analyses revealed thermal redistribution of conformational components. FTIR showed non-monotonic changes in β-sheet, α-helix, β-turn, and random-coil contributions, whereas circular dichroism demonstrated retention of the β-sheet-rich structural framework with a modest increase in estimated antiparallel β-sheet content. Quantitative X-ray powder diffraction analysis showed an increase in relative crystallinity from 45.41% in untreated RSF to 52.33% and 70.85% after treatment at 50 and 100 °C, respectively, with a corresponding decrease in the amorphous fraction. Dynamic light scattering revealed an increase in hydrodynamic size and polydispersity, while microscopic analyses showed the formation of larger, more interconnected, and morphologically heterogeneous assemblies at elevated temperatures. Surface roughness also increased most prominently in the 100 °C-treated sample. These findings demonstrate that thermal treatment promotes coupled changes in the local molecular environment, hydrophobic exposure, secondary-structure distribution, molecular ordering, and higher-order assembly of RSF, providing an integrated description of its thermally induced structural evolution.

## Abstract 2

Regenerated silk fibroin (RSF) is a protein biomaterial whose molecular conformation, aggregation behavior, and functional properties are strongly influenced by processing conditions.

## Graphical abstract

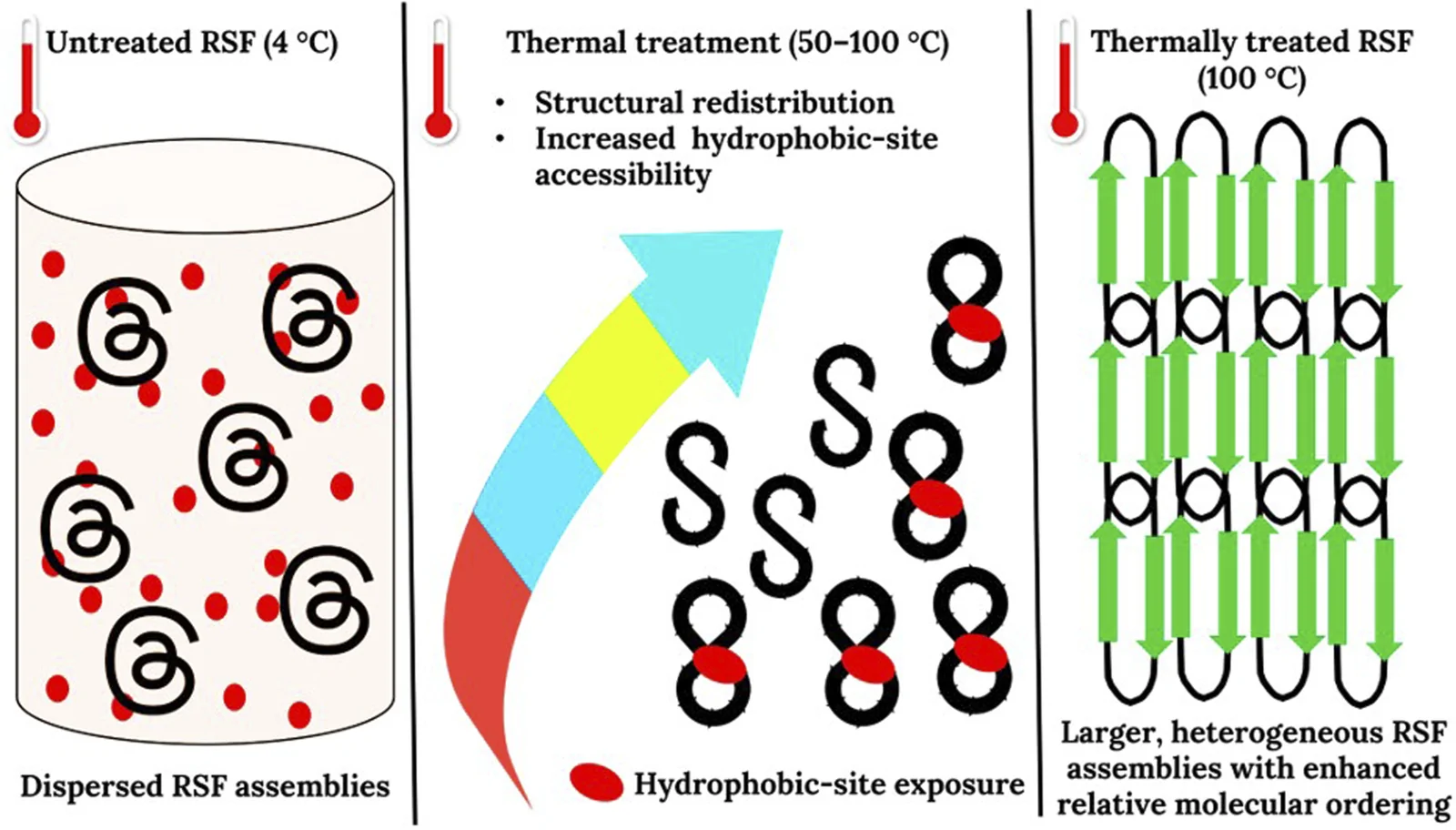

## 1. Introduction

Silk fibroin (SF), the structural protein extracted from *Bombyx mori* silkworm cocoons, has emerged as a versatile and sustainable biomaterial due to its biocompatibility, mechanical robustness, thermal stability, and tunable biodegradability.^1^ The molecular structure of SF consists of a heavy chain, a light chain, and a P25 glycoprotein that assembles into hierarchical architectures. Depending on environmental conditions and processing methods, SF can adopt distinct structural forms: Silk I (metastable, hydrated), Silk II (β-sheet-rich, crystalline), and Silk III (helical, interfacial).^1^ Among these, Silk II is particularly important for biomaterial applications, as its β-sheet content imparts superior mechanical strength and thermal resistance. Consequently, controlling the structural transition of SF, especially from random coil or Silk I toward Silk II, has significant implications in biomedical engineering, electronics, and nanofabrication.^1,2^ In addition to its technological importance, understanding the molecular processes governing SF self-assembly and aggregation is essential for controlling material performance, stability, and reproducibility during processing.

The self-assembly and aggregation behavior of SF is strongly influenced by external stimuli such as pH, ionic strength, dehydration, molecular crowding, and thermal treatment.^3^ Temperature plays an important role in modulating conformational transitions and intermolecular interactions. Several studies have demonstrated that thermal treatment or incubation can promote SF aggregation, β-sheet formation, and morphological reorganization.^1,4,5^ For instance, regenerated silk fibroin (RSF) incubated at 25–75 °C showed increased β-sheet content and morphological reorganization, with protofibrillar structures becoming more prominent at elevated temperatures.^4^ Similarly, casting SF membranes at progressively higher temperatures (20–80 °C) resulted in denser nanofibril arrangements and increased Silk II crystallinity.^1^ Optical spectroscopy studies have also demonstrated that temperature-dependent changes in regenerated *B. mori* SF can be monitored through intrinsic fluorescence and circular dichroism, with changes in Tyr/Trp fluorescence and secondary structure observed during heating over 0–100 °C.^6^ These observations establish the sensitivity of aromatic-residue fluorescence to the molecular environment of RSF during conformational remodeling. More recently, kinetic analysis demonstrated that RSF self-assembly can involve secondary nucleation processes, highlighting the complexity of the aggregation pathways involved in silk assembly.^7^ Molecular dynamics simulations further support the greater thermal and pressure stability of Silk II relative to Silk I, emphasizing the importance of β-sheet organization in silk-based materials.^8^

The structural response of SF to heat is also dependent on the treatment conditions and physical state of the material. Kweon *et al.* demonstrated that regenerated *Antheraea pernyi* SF films undergo temperature and time-dependent conformational and crystalline changes, with pronounced β-sheet-associated changes occurring at substantially higher temperatures than those investigated in the present aqueous RSF system. This distinction is important because structural changes observed during high-temperature treatment of regenerated SF films cannot be directly assumed to represent the behavior of aqueous *B. mori* RSF under moderate thermal conditions.^9^ Beyond thermal treatment, SF assembly is also influenced by cosolvents, ions, glucose units, and macromolecular crowding agents. Ethanol and methanol can accelerate β-sheet aggregation and fibril formation,^2,10,11^ while Ca^2+^ ions can stabilize intermediate α-helical conformations during the transition toward β-sheet-rich assemblies.^12^ Macromolecular crowding can further accelerate SF fibrillization and β-sheet crystallization.^13^ Conversely, curcumin and β-cyclodextrin have been reported to inhibit ethanol-induced fibrillization and maintain SF in a random-coil state.^2,11^ Dry and wet heat treatments, such as exposure to 180 °C, can also produce different structural outcomes in SF sponges, further demonstrating the dependence of SF remodeling on the surrounding physicochemical environment.^3^ Despite these advances, relationships between local molecular changes, secondary-structure redistribution, aggregate growth, molecular ordering, and higher-order morphology during moderate-temperature thermal treatment remain insufficiently established.

The current study systematically investigates the temperature-dependent aggregation behavior and structural evolution of regenerated silk fibroin (RSF) using a multiparametric approach that integrates intrinsic and extrinsic fluorescence with spectroscopic, structural, and morphological characterization. Intrinsic fluorescence and ultraviolet-visible (UV-vis) absorption measurements are combined with time-correlated single-photon counting (TCSPC) fluorescence lifetime measurements to examine temperature-dependent changes in aromatic-residue fluorescence behavior and dynamics. These measurements are complemented by extrinsic probes, including Thioflavin T (ThT), 8-anilino-1-naphthalenesulfonic acid (ANS), and Congo Red, and further correlated with circular dichroism (CD), Fourier-transform infrared spectroscopy (FTIR), X-ray powder diffraction (XRPD), dynamic light scattering (DLS), scanning electron microscopy (SEM), atomic force microscopy (AFM), and fluorescence microscopy. The RSF samples were thermally treated across 50–100 °C, with intrinsic fluorescence providing the temperature-dependent response across the investigated range. For complementary structural and morphological analyses, untreated RSF and selected thermally treated states at 50 and 100 °C were examined to represent the lower and upper boundaries of the applied thermal range. This strategy enables the temperature-dependent spectroscopic response to be evaluated together with corresponding changes in secondary structure, molecular ordering, aggregate size, and morphology, while avoiding the assumption that every structural parameter changes continuously with temperature. Rather than proposing a new elementary aggregation mechanism, the present study establishes experimentally supported relationships among aromatic-residue fluorescence behavior, fluorescence lifetime, hydrophobic exposure, β-sheet-associated structural changes, aggregate growth, and morphological reorganization during thermal treatment. By integrating measurements that probe different structural scales, the study provides a more comprehensive description of RSF thermal remodeling than individual spectroscopic or morphological measurements, enabling relationships between local molecular-environment changes and higher-order structural organization to be evaluated within the same thermal-treatment framework.

## 2. Materials and methods

### 2.1. Chemicals

All reagents and chemicals used in this study were of analytical grade. Regenerated silk fibroin (RSF), derived from domesticated *Bombyx mori* silkworm cocoons, was procured from Sigma-Aldrich (Product No. 5154). The RSF was supplied as an aqueous solution at a concentration of 50 mg ml^−1^, with an average molecular weight (*M*_w_) of approximately 100 kDa, as specified by the manufacturer. Fluorescent probes, including 8-anilino-1-naphthalenesulfonic acid (ANS; ≥96%, A1028), Thioflavin T (ThT; T3516), and Congo Red (CR; C6767), were obtained from Sigma-Aldrich (St. Louis, MO, USA). Muscovite mica substrates used for atomic force microscopy (AFM; AFM-71856-03) were also obtained from Sigma-Aldrich.

### 2.2. Thermal modification of regenerated silk fibroin

Regenerated silk fibroin (RSF) solution (Product No. 5154; average molecular weight ≈100 kDa; Sigma-Aldrich), derived from domesticated *Bombyx mori* silkworm cocoons, was used as the starting material for all experiments. The stock RSF solution (50 mg ml^−1^) was diluted with Milli-Q water to a final working concentration of 10 mg ml^−1^, following previously reported protocols.^12^ Thermal modification of RSF was performed by incubating separate aliquots of the diluted RSF solution in a thermostatically controlled water bath at 50, 60, 70, 80, 90, and 100 °C, respectively, for 3 h at each temperature. Each temperature condition was applied independently using fresh RSF aliquots to avoid cumulative or stepwise heating effects. A single water bath was used for all experiments, with the bath temperature adjusted sequentially to the desired set point and allowed to equilibrate before sample incubation. Untreated RSF samples maintained at 4 °C were used as experimental controls. Based on the temperature-dependent intrinsic fluorescence response, untreated RSF and thermally treated RSF at 50 and 100 °C were selected as representative conditions for complementary structural and morphological characterization, corresponding to the lower and upper boundaries of the investigated temperature range. For the highest temperature condition (100 °C), additional time-resolved measurements were conducted to probe aggregation kinetics. During this incubation, aliquots were withdrawn at 30 min intervals and analyzed using extrinsic fluorescent probes, including ThT, ANS, and Congo Red, to monitor the temporal evolution of β-sheet formation and hydrophobic surface exposure.

### 2.3. Spectral data processing

Raw fluorescence, UV-visible, FTIR, and XRPD data obtained from untreated and thermally modified RSF were processed using SpectraGryph v1.2.16.1. For intrinsic fluorescence measurements, the region of interest (ROI) was selected from 300 to 600 nm, and the spectra were analyzed to identify characteristic emission features.^14^ Fluorescence spectra obtained from the Tecan Spark multimode microplate reader (Männedorf, Switzerland), as well as FTIR and XRPD spectra, were subsequently processed using the same software according to the respective analysis procedures described in the corresponding sections. Fluorescence lifetime data were analyzed using instrument-associated lifetime analysis software, and biexponential decay fitting was used to obtain the fluorescence lifetime components and intensity-weighted average lifetime. Graphical presentation and statistical analyses were performed using GraphPad Prism version 8.0 and OriginPro 2026.

### 2.4. Statistical analysis

The mean and standard deviation (SD) of the fluorescence spectroscopy and DLS measurements were calculated using Microsoft Excel 2019. Statistical comparisons were performed using Brown–Forsythe and Welch one-way ANOVA, which does not require the assumption of equal variances among groups. For fluorescence spectroscopy data, significant differences among groups were further evaluated using the Games–Howell multiple-comparisons test. For DLS data, pairwise comparisons were performed using unpaired *t*-tests with Welch's correction. Statistical significance was indicated as **p ≤ 0.01 and ****p ≤ 0.0001. All data were plotted using GraphPad Prism version 8.0 and OriginPro 2026.

### 2.5. Additional experimental details

Detailed experimental procedures for the characterization techniques, including fluorescence spectroscopy, UV-visible spectroscopy, lifetime measurements, ThT, ANS, Congo Red assays, FTIR, CD, SEM, fluorescence microscopy, AFM, XRPD, and DLS, are provided in the SI.

## 3. Results and discussion

### 3.1. Temperature-dependent spectroscopic remodeling of RSF

The intrinsic fluorescence spectra of RSF, excited at 285 nm, exhibited a progressive increase in emission intensity with increasing thermal treatment from 50 to 100 °C, while the overall spectral profile and emission maximum around 320 nm remained essentially unchanged (Fig. 1a). The fluorescence intensity at 320 nm showed the same temperature-dependent trend, with a highly significant increase across the investigated conditions (****p < 0.0001; Fig. 1b). The emission in this region is associated with the intrinsic aromatic residues of the protein, particularly tyrosine (Tyr) and tryptophan (Trp), whose fluorescence is sensitive to their local molecular environment. Wray *et al.* demonstrated that intrinsic fluorescence could distinguish processing-dependent changes in silk fibroin,^15^ while Yang *et al.* showed that optical spectroscopy can be used to follow structural changes in regenerated *Bombyx mori* silk fibroin in solution.^6^ The present results are consistent with these observations, as thermal treatment produced a measurable change in fluorescence intensity without a substantial shift in the emission maximum. This suggests that the treatment primarily modifies the photophysical environment of the existing aromatic residues rather than producing a distinctly different fluorescent species. Such changes are compatible with the known ability of fibroin to undergo gradual conformational rearrangement during self-assembly. Dubey *et al.* described fibroin self-assembly as a progressive process involving redistribution of secondary structural states,^12^ while Xiao *et al.* showed that heat-induced treatment can drive fibroin toward larger, more organized assemblies.^5^ The present fluorescence measurements demonstrate a progressive temperature-dependent change in the aromatic-residue fluorescence response across the applied 50–100 °C range, providing a local spectroscopic indicator of the thermal response.

**Fig. 1:**
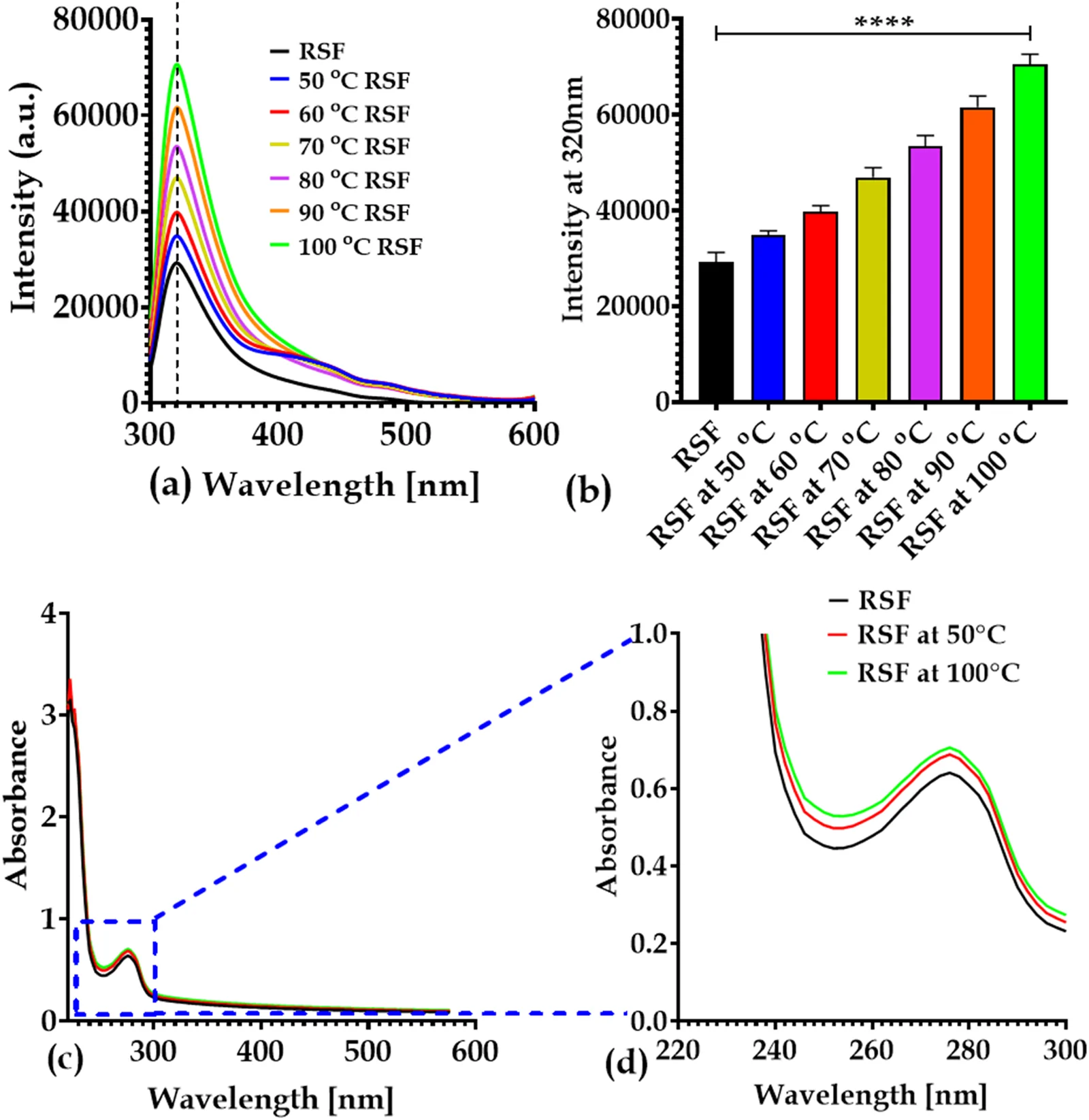
Intrinsic fluorescence and absorbance spectra of RSF following thermal modification. (a) Fluorescence emission spectra of untreated RSF and RSF thermally modified at 50–100 °C, excited at 285 nm; (b) fluorescence intensity at 320 nm. Data are presented as mean ± SD (*n* = 3). Statistical significance was assessed using Brown–Forsythe and Welch one-way ANOVA followed by Games–Howell multiple-comparison test (****p < 0.0001); and (c and d) UV-visible absorbance spectra of untreated and thermally modified RSF (50 and 100 °C) recorded over 220–600 nm.

The fluorescence changes were further examined by UV-visible absorption spectroscopy to determine whether thermal treatment also affected the absorption characteristics of the protein (Fig. 1c and d). Untreated and thermally modified RSF exhibited a similar near-UV absorption profile with a maximum around 276–280 nm. Treatment at 50 and 100 °C increased the absorbance intensity, particularly at 100 °C, while the position of the principal absorption feature and the overall spectral profile remained largely unchanged. This near-UV absorption is associated with electronic transitions of aromatic amino acid residues and is consistent with the optical behavior reported for silk fibroin by Perotto *et al.* who showed that the optical properties of regenerated silk fibroin depend on its molecular and structural characteristics,^16^ and by Okahisa *et al.* who reported a characteristic UV absorption feature near 275 nm for silk fibroin.^17^ The increase in absorbance observed here, together with the increase in fluorescence intensity, indicates a temperature-dependent alteration in the optical response of RSF, while the largely unchanged spectral profile provides no evidence of the formation of a new dominant absorbing species. The sensitivity of aromatic-residue absorption to the local molecular environment provides a possible basis for this response; in particular, Fornander *et al.* demonstrated that the UV transition of Tyr is affected by its surrounding environment, including polarity and hydrogen-bonding interactions.^18^ During thermal treatment, the samples were maintained under closed conditions to minimize evaporation-related changes in protein concentration. Because changes in absorbance and scattering can influence measured fluorescence intensity, the present fluorescence and UV-visible measurements do not by themselves establish whether the observed changes arise from altered solvent accessibility, intermolecular interactions, changes in scattering, or other effects associated with thermal treatment. Therefore, the fluorescence response was further evaluated using time-resolved measurements.

TCSPC fluorescence lifetime measurements were performed for untreated RSF and the representative 50 and 100 °C conditions to determine whether a change in fluorescence decay behavior accompanied the temperature-dependent change in steady-state fluorescence. The decay profiles of all three samples were adequately described by a biexponential model, with the fitted decay curves, residuals, autocorrelation profiles, and complete fitting data provided in the SI (Fig. S1–S7 and Table S1). The shorter lifetime component (*τ*_1_) increased from 1.30869 ± 0.00718 ns for untreated RSF to 1.34890 ± 0.00775 ns and 1.37702 ± 0.00780 ns following treatment at 50 and 100 °C, respectively, whereas the longer component (*τ*_2_) remained relatively constant at 4.51702 ± 0.03870, 4.48690 ± 0.03525, and 4.47622 ± 0.03056 ns, respectively. The intensity-average lifetime also increased from 1.86298 ns for untreated RSF to 1.91907 ns and 2.00426 ns after treatment at 50 and 100 °C, respectively. The corresponding reduced *χ*^2^ values were 1.12916, 1.30708, and 1.09973, indicating comparable fitting quality across the three samples. The increases in the shorter lifetime component and intensity-average lifetime indicate that the excited-state dynamics of the intrinsic fluorophore population change with thermal treatment. Sahoo *et al.* demonstrated that intrinsic Trp fluorescence lifetime can provide complementary information on protein aggregation and changes in the fluorophore environment,^19^ while Zhdanova *et al.* showed that Tyr fluorescence intensity and lifetime can respond to relatively subtle changes in protein conformation and local environment.^20^ In the present RSF system, the lifetime results therefore provide independent evidence of changes in the excited-state dynamics of the intrinsic fluorophore population accompanying thermal treatment, thereby complementing the steady-state fluorescence measurements. Importantly, fluorescence lifetime is an intensity-independent parameter and is not directly determined by fluorophore concentration; therefore, the observed lifetime changes provide complementary evidence for altered fluorophore photophysics accompanying thermal treatment, rather than relying solely on changes in steady-state fluorescence intensity. These observations provide a spectroscopic basis for examining whether the changes in aromatic-residue fluorescence are associated with the changes in hydrophobic-site accessibility, secondary-structure distribution, aggregate size, and morphology described in the following sections.

### 3.2. Thermal modulation of hydrophobicity and β-sheet-associated structures

ThT fluorescence was first used to examine the development of ordered, dye-accessible structures during thermal treatment of RSF. Untreated RSF showed a broad emission band centered at approximately 490–500 nm, which increased in intensity after treatment at 50 and 100 °C, with the highest response observed at 100 °C, while the emission maximum remained essentially unchanged (Fig. 2a). The absence of an appreciable spectral shift suggests that thermal treatment predominantly changes the extent of ThT association rather than producing a substantially different emissive species. The time-dependent response at 490 nm also increased progressively during 180 min of treatment at 100 °C (Fig. 2b), indicating that the formation of ThT-accessible structures continues during thermal exposure. ThT fluorescence is widely used to monitor the formation of ordered β-sheet-associated assemblies because its fluorescence increases upon association with suitable repetitive protein surfaces; however, the magnitude of the response depends on the structural characteristics of the binding sites and does not, by itself, establish amyloid fibril formation.^21^ In silk fibroin, increased ThT fluorescence has similarly been associated with the progression of self-assembly and β-sheet formation under different aggregation conditions.^12,13^ Heat-induced silk assembly has also been reported to involve rapid β-sheet formation together with nucleation-dependent aggregation,^5^ while Qian *et al.* showed that heating can increase the β-sheet content of silk fibroin.^22^ Thus, the increased ThT response is consistent with greater accessibility of ordered β-sheet-associated structures, but does not by itself establish the specific aggregation mechanism or a classical amyloid cross-β architecture.

**Fig. 2:**
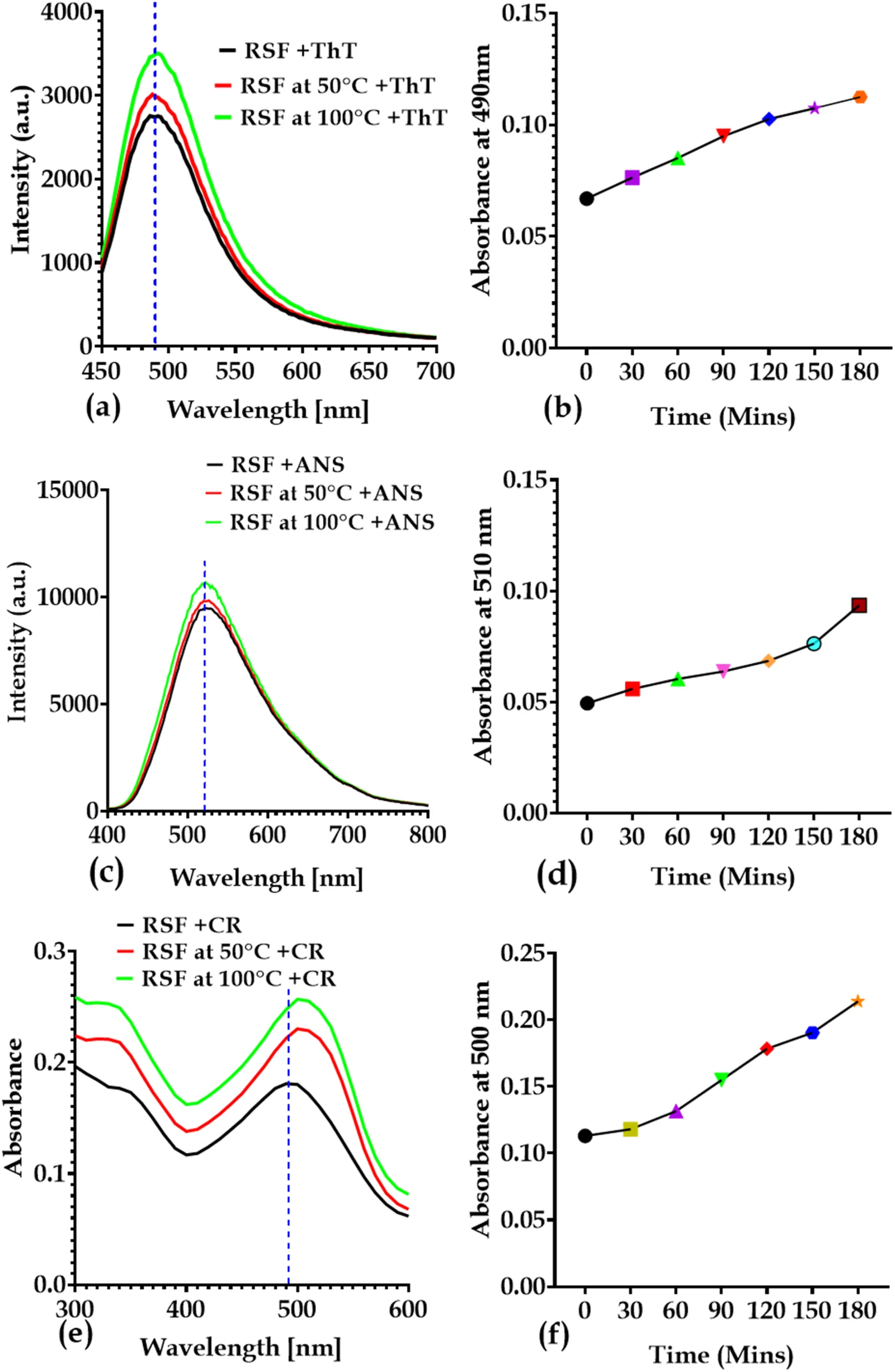
Amyloid-sensitive dye assays of untreated and thermally modified RSF (50 °C and 100 °C). (a) ThT fluorescence spectra recorded at 400 nm excitation; (b) time-dependent ThT absorbance at 490 nm for RSF thermally modified at 100 °C; (c) ANS fluorescence spectra recorded at 350 nm excitation; (d) time-dependent ANS absorbance at 510 nm for RSF thermally modified at 100 °C; (e) Congo Red (CR) absorption spectra recorded over 300–600 nm; and (f) time-dependent CR absorbance at 500 nm for RSF thermally modified at 100 °C.

The changes detected by ThT were complemented by ANS fluorescence, which provides information on the accessibility of hydrophobic regions. Untreated RSF exhibited a broad ANS emission band near 520 nm, whereas thermally treated samples showed increased fluorescence intensity, particularly after treatment at 100 °C, without a substantial displacement of the emission maximum (Fig. 2c). The time-dependent ANS response at 510 nm also increased during 180 min of treatment at 100 °C (Fig. 2d). These results indicate an increase in ANS-accessible hydrophobic sites during thermal treatment. ANS fluorescence is sensitive to the hydrophobicity and local mobility of its binding environment, and studies using silk fibroin have demonstrated that ANS can report protein sites with different degrees of solvent accessibility.^23^ Previous investigations have also shown that changes in silk fibroin self-assembly can be accompanied by alterations in hydrophobic interactions and secondary structure.^11–13^ Thus, the increased ANS response supports greater accessibility of hydrophobic sites during thermal treatment, while hydration-shell disruption, chain mobility, and specific intermolecular hydrophobic interactions remain possible but unconfirmed contributors.

Congo Red (CR) measurements provided an additional probe of the structural changes detected by ThT and ANS. Untreated RSF displayed relatively low absorbance around 500 nm, whereas thermally treated samples showed increased CR absorbance with a more pronounced response after treatment at 100 °C (Fig. 2e). The corresponding time-dependent measurements at 500 nm increased progressively during thermal exposure (Fig. 2f), indicating an increasing population of CR-accessible structures. Previous work has demonstrated increased CR binding during silk fibroin aggregation induced by ethanol, together with changes in secondary structure and formation of larger assemblies.^11^ Similarly, Osawa *et al.* observed ThT- and CR-positive, fibrillar silk fibroin after ethanol treatment, while emphasizing that such dye responses describe amyloid-like structural properties and do not necessarily imply biological amyloidogenicity.^24^ Thus, the enhanced CR response in the present study is interpreted as evidence of increased accessibility of ordered, CR-binding structures rather than definitive evidence of amyloid fibrils. The three dye assays provide complementary evidence of thermal RSF remodeling: increased ThT and CR responses indicate greater accessibility of ordered structures, while increased ANS fluorescence suggests greater hydrophobic-site accessibility. These findings are consistent with reported changes in silk fibroin self-assembly.^5,11–13,22^ Because the dye assays alone do not establish the underlying mechanism, these observations are interpreted alongside FTIR, CD, XRPD, and DLS analyses.

### 3.3. Secondary structural redistribution and molecular organization of RSF

FTIR amide-I deconvolution (1580–1720 cm^−1^) showed temperature-dependent redistribution of fitted secondary-structure components in RSF (Fig. 3a–g and Table S2). Untreated RSF was β-sheet-rich (75.34%), with 17.34% α-helix, 3.23% β-turn, and 4.09% random coil. At 50 °C, α-helix increased slightly to 20.65%, whereas β-sheet decreased to 48.67%; β-turn and random coil increased to 23.35% and 7.33%, respectively. At 100 °C, α-helix decreased to 8.43%, while β-sheet increased relative to 50 °C to 54.00%; β-turn and random coil reached 24.29% and 13.28%, respectively. Thus, the FTIR data indicate structural redistribution rather than a uniform increase in molecular order, as the higher random-coil contribution at 100 °C coexisted with increased β-sheet-associated signal relative to 50 °C. A broadly similar intermediate-temperature pattern was reported by León-Heredia *et al.*, who observed increased α-helix and reduced β-sheet near 55 °C in squid myofibrillar proteins, followed by increased β-sheet at higher temperatures.^25^ Farnsworth *et al.* likewise reported temperature-associated α-helix increase with compensatory β-sheet decrease in α-crystallin,^26^ while Dubey *et al.* identified a transient α-helical intermediate during calcium-mediated fibroin self-assembly.^12^ Conversely, Qian *et al.* reported increased β-sheet content after heating silk fibroin to 95 °C, in agreement with the relative β-sheet increase in RSF at 100 °C *versus* 50 °C. Nevertheless, because the 100 °C β-sheet contribution remained below the untreated value, the data indicate partial redistribution rather than recovery of the original β-sheet population.^22^ The transition from random-coil to β-sheet-rich fibroin during ethanol-induced assembly reported by Dubey *et al.* further emphasizes that fibroin secondary structure depends on the applied stimulus and treatment conditions.^11^

**Fig. 3:**
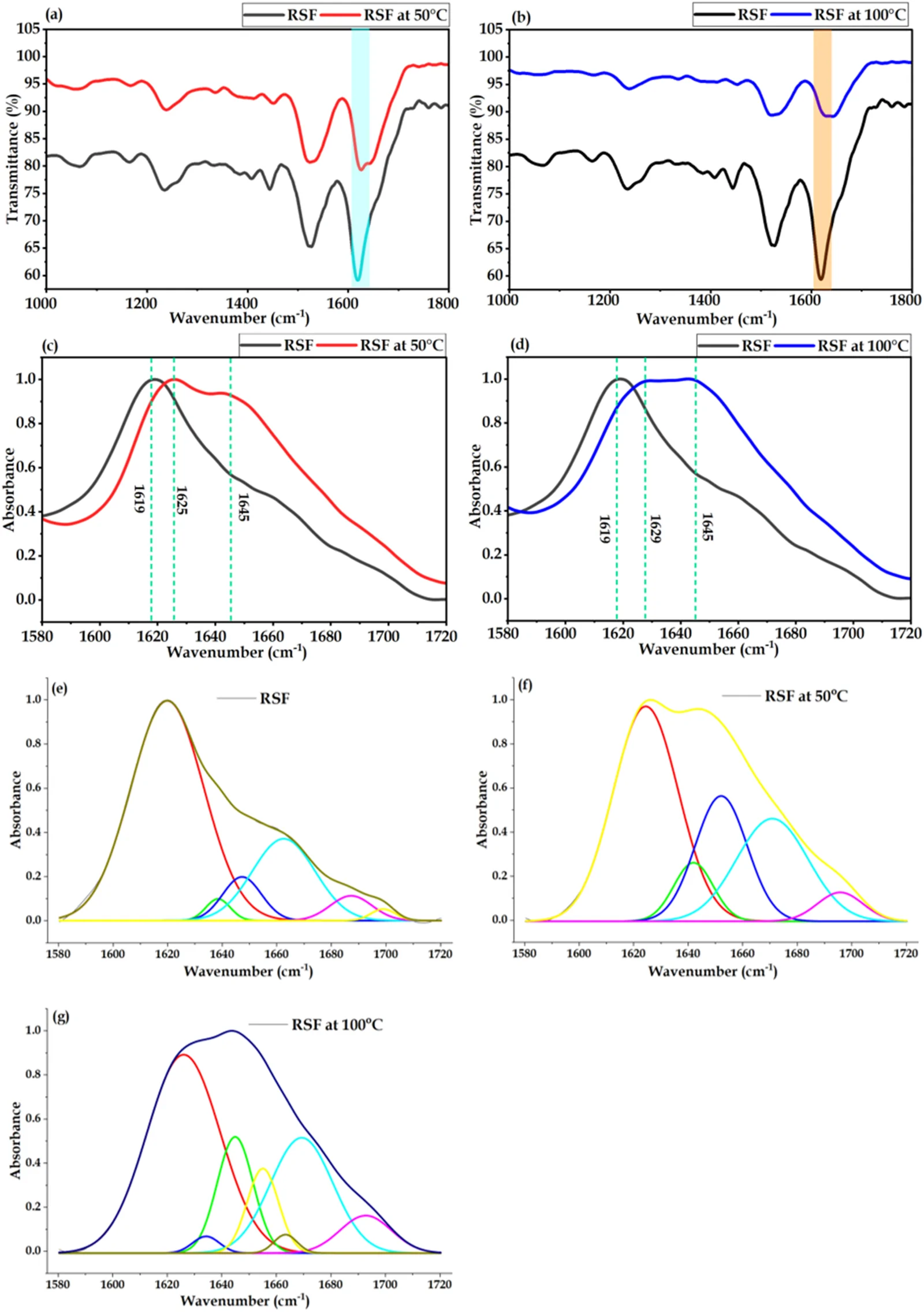
FTIR spectral analysis and Gaussian deconvolution of untreated and thermally modified RSF. (a) FTIR spectra of untreated RSF and RSF treated at 50 °C (1000–1800 cm^−1^); (b) FTIR spectra of untreated RSF and RSF treated at 100 °C (1000–1800 cm^−1^); (c) normalized amide I band spectra of untreated RSF and RSF treated at 50 °C; (d) normalized amide I band spectra of untreated RSF and RSF treated at 100 °C; (e) Gaussian deconvolution of the amide I band of untreated RSF; (f) Gaussian deconvolution of the amide I band of RSF treated at 50 °C; and (g) Gaussian deconvolution of the amide I band of RSF treated at 100 °C.

Far-UV CD provided complementary solution-state evidence that the overall β-sheet-rich RSF framework was retained after thermal treatment (Fig. 4a). The negative band near 216–218 nm persisted in all conditions, while BeStSel estimated antiparallel β-sheet contents of 41.5%, 43.7%, and 45.3% for untreated RSF and RSF treated at 50 and 100 °C, respectively. The estimated helical fraction decreased from 3.4% to 1.7% and was not detected at 100 °C; turn content remained comparable (13.0%, 11.7%, and 13.0%), and the remaining contribution was approximately 42%, as shown in (Table S3 and Fig. S8–S10). Therefore, CD showed modest β-sheet enrichment with heating, whereas FTIR revealed a non-monotonic redistribution of fitted amide-I components. The CD trend is qualitatively consistent with the high-temperature loss of α-helix and increase in β-sheet observed by.^22,25^ The reduced helical contribution at 100 °C also agrees with the FTIR result. However, the absolute values derived by FTIR and CD should not be directly compared, because ATR-FTIR was performed on lyophilized material, whereas CD was measured in aqueous solution, and the methods apply different spectral assignments and deconvolution models. Dong *et al.* similarly showed that FTIR and CD may produce different numerical secondary-structure estimates while yielding a consistent overall conclusion regarding β-sheet-rich protein structure.^27^ Therefore, the complementary results show that heating alters the distribution of RSF secondary-structure components while maintaining a β-sheet-rich global framework; they do not independently establish a continuous transition pathway, specific aggregate architecture, or particular intermolecular mechanism.

**Fig. 4:**
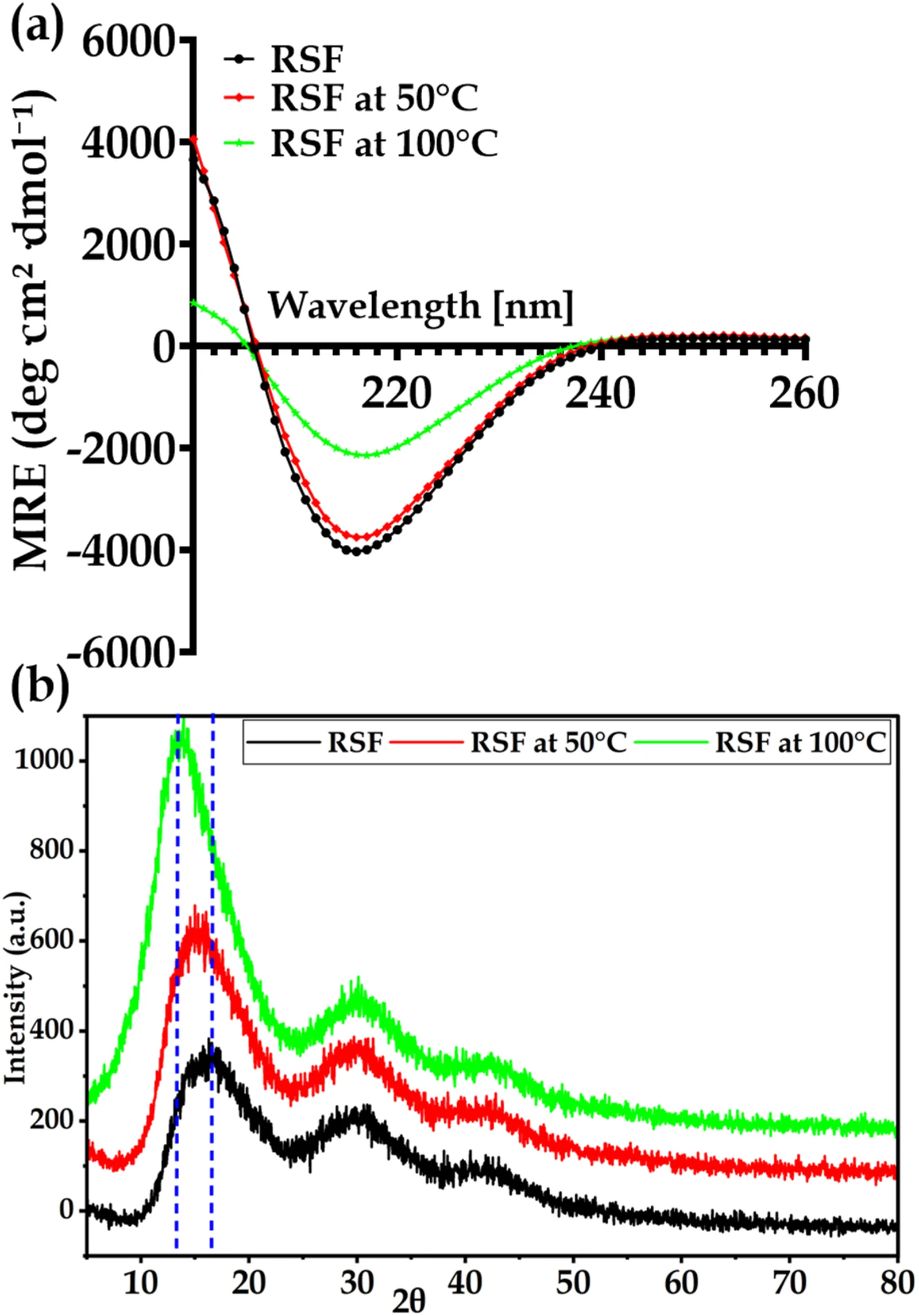
Secondary structural and crystalline characteristics of untreated and thermally modified RSF at 50 °C and 100 °C. (a) Circular dichroism (CD) spectra recorded in the far-UV region (190–260 nm); and (b) X-ray powder diffraction (XRPD) patterns recorded over the 2*θ* range.

XRPD was used to determine whether the temperature-associated secondary-structure redistribution detected by FTIR and CD was accompanied by altered solid-state molecular ordering in lyophilized RSF (Fig. 4b). All samples showed broad, partially overlapping diffraction features, consistent with a mixed crystalline–amorphous organization rather than a single sharply resolved crystalline phase. However, deconvolution of the XRPD profiles showed a progressive increase in the crystalline contribution with thermal treatment (Fig. S11). The crystalline integrated area increased from 2934.80 arbitrary units in untreated RSF to 4200.12 and 6555.91 arbitrary units after treatment at 50 and 100 °C, respectively. Accordingly, the crystallinity index increased from 45.41% in untreated RSF to 52.33% at 50 °C and 70.85% at 100 °C, while the amorphous fraction decreased from 54.59% to 47.67% and 29.15%, respectively (Table S4). These quantitative results demonstrate increased relative molecular ordering following thermal treatment. Comparable increases in crystalline or β-sheet-associated structure have been reported after serine-directed chemical modification of silk fibroin by Liu *et al.*,^28^ alcohol post-treatment of regenerated silk fibroin films by Puerta *et al.*,^29^ and ethanol-coagulation-mediated RSF fiber formation by Zhang and Pan.^30^ In contrast, Wu *et al.* showed that regenerated fibroin and native degummed silk possess distinct diffraction profiles and degrees of organization,^31^ while Yaiphabi *et al.* reported broad diffraction maxima for regenerated silk fibroin films, supporting the semicrystalline and structurally heterogeneous nature of regenerated fibroin.^32^ Zhao *et al.* further demonstrated that the stability and diffraction signatures of Silk I and Silk II depend on the initial structural state and processing conditions.^2^ Therefore, although the present results agree with the increased antiparallel β-sheet contribution estimated by CD and the enhanced ThT and Congo Red responses, they support an increase in relative crystalline ordering rather than an unambiguous Silk I-to-Silk II phase transition or a specific intermolecular mechanism.

### 3.4. Morphological and hydrodynamic evolution of thermally treated RSF

SEM was employed to examine temperature-associated changes in the surface morphology of RSF (Fig. 5). Untreated RSF (Fig. 5a) exhibited a heterogeneous porous microstructure comprising thin sheet-like/lamellar regions, interconnected fibrillar features, globular structures, and relatively open pore regions. At 50 °C (Fig. 5b), the porous architecture was retained, while the lamellar/sheet-like features appeared more continuous and interconnected, with localized globular structures within and around the surface voids. At 100 °C (Fig. 5c), denser clustered regions, thicker sheet-like structures, and ribbon-like features became more prominent, although the porous framework remained evident. Representative lamellar/sheet-like, fibrillar/ribbon-like, and globular features are indicated by arrows in (Fig. 5a–c). These observations indicate temperature-associated surface reorganization and apparent densification without large-scale collapse of the porous architecture, consistent with previous observations of thermally reorganized silk fibroin structures.^3,33,34^

**Fig. 5:**
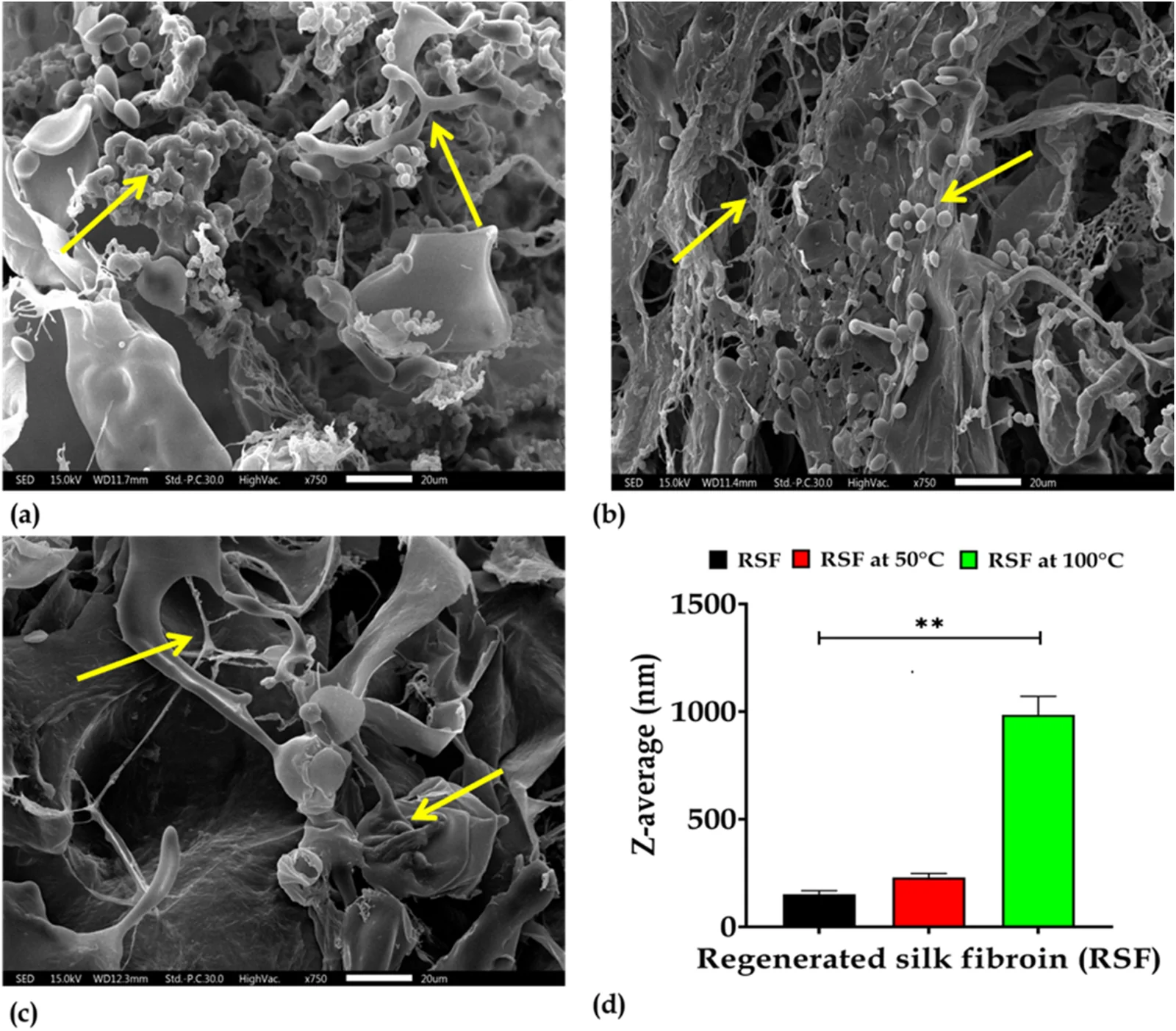
Morphological and hydrodynamic characterization of untreated and thermally modified RSF. (a) Scanning electron microscopy (SEM) image of untreated RSF; (b) RSF treated at 50 °C; (c) RSF treated at 100 °C. Representative lamellar/sheet-like, fibrillar/ribbon-like, and globular morphological features are indicated by arrows. All SEM images were acquired at ×750 magnification with a 20 µm scale bar. (d) *Z*-average hydrodynamic diameter of untreated RSF and thermally modified RSF (50 °C and 100 °C) determined by dynamic light scattering (DLS). Data are presented as mean ± standard deviation from three independent measurements (*n* = 3). Statistical significance was assessed using Brown–Forsythe and Welch one-way ANOVA, followed by pairwise unpaired *t*-tests with Welch's correction (**p < 0.01).

To determine whether changes in dispersed RSF assemblies accompanied these morphological changes, DLS was subsequently performed (Fig. 5d). The *Z*-average hydrodynamic diameter increased from 131.8–161.6 nm for untreated RSF to 208.4–243.7 nm at 50 °C and 893.0–1062 nm at 100 °C, accompanied by an increase in PDI from 0.264–0.294 to 0.355–0.398 and 0.414–0.547, respectively. Thus, thermal treatment increased both hydrodynamic size and dispersity, indicating progressively larger and more heterogeneous RSF assemblies rather than a single uniform population. The overall difference among groups was significant (**p < 0.01, Brown–Forsythe and Welch one-way ANOVA), which was selected because it does not assume equal variances; pairwise comparisons were performed using unpaired *t*-tests with Welch's correction. Individual measurements and representative intensity-weighted distributions are provided in (Table S5 and Fig. S12–S14). The presence of multiple hydrodynamic populations agrees with previous observations for silk fibroin solutions,^35^ while the distinction between hydrated DLS species and dried SEM structures should be considered when comparing the two measurements.^36^ The temperature-dependent increase observed here also differs from the particle-size decrease reported in a thermally treated β-lactoglobulin-gum arabic system, emphasizing the system-dependent nature of thermal remodeling.^37^ The increased DLS size and PDI, therefore, provide complementary evidence for thermally associated RSF assembly and heterogeneity, but do not by themselves define the underlying intermolecular interactions.

The increase in assembly size and heterogeneity was further visualized by fluorescence microscopy following incubation with 10 µM ThT (Fig. 6). Untreated RSF (Fig. 6a) showed predominantly small, discrete fluorescent structures, whereas the 50 °C sample (Fig. 6b) exhibited larger, elongated and interconnected assemblies; at 100 °C (Fig. 6c), more densely clustered and heterogeneous structures were observed across the DAPI, FITC and TRITC channels. These spatial changes are consistent with the DLS-derived increase in assembly size and heterogeneity. Previous studies have demonstrated that intrinsic tryptophan fluorescence can visualize RSF self-assembly, although fibrillar morphology cannot be assigned to all RSF aggregates,^38^ while the intrinsic autofluorescence of regenerated silk fibroin across multiple optical channels has also been established.^39,40^ Similar microscopy-based evidence of larger and heterogeneous assemblies has been reported for β-casein/β-lactoglobulin and HSA/BSA,^14,41^ as well as for peroxynitrite- and dopamine-modified hemoglobin, where complementary analyses indicated predominantly non-fibrillar assemblies.^42,43^

**Fig. 6:**
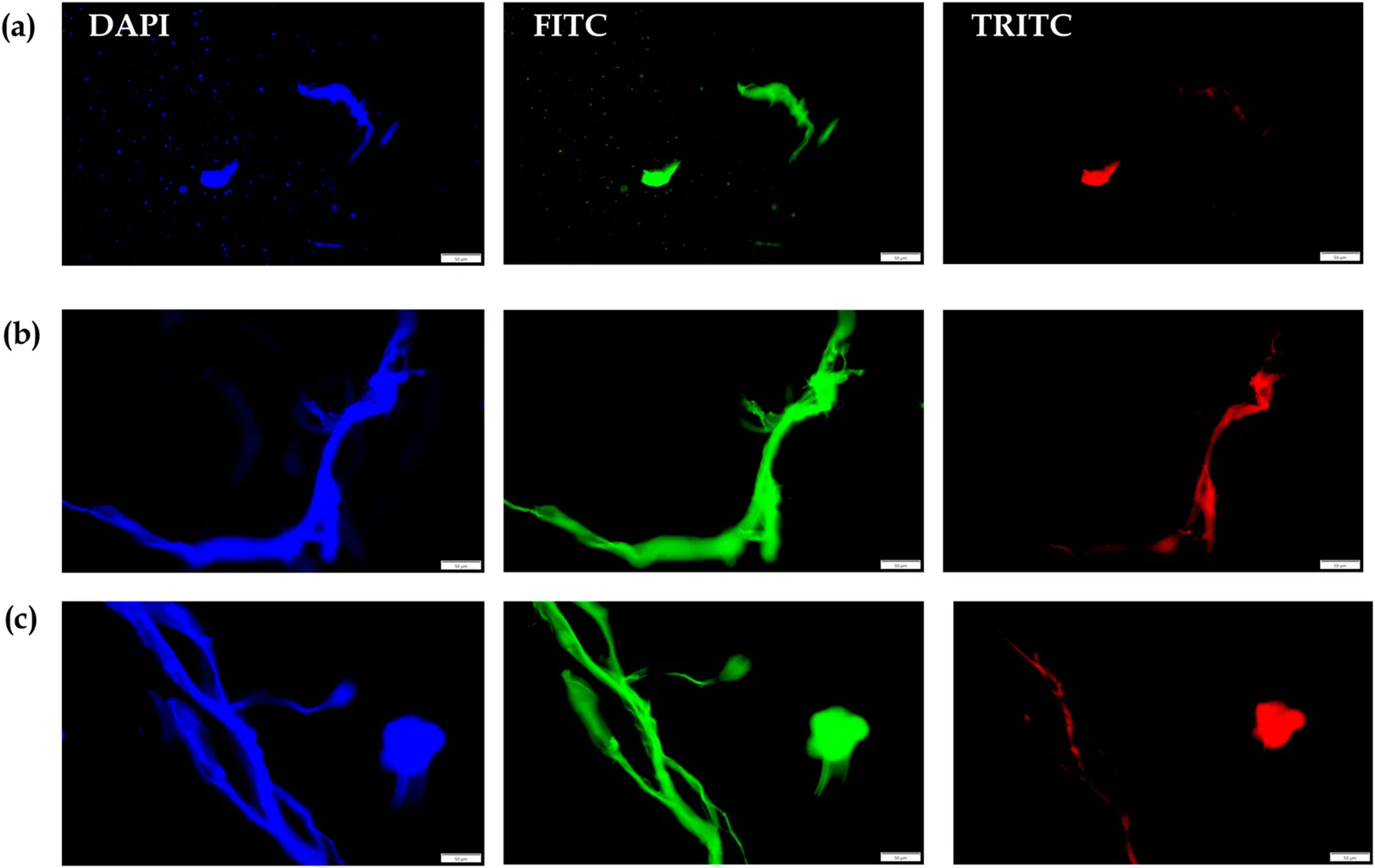
Fluorescence microscopy images of untreated and thermally modified RSF. (a) Untreated RSF, (b) RSF heated at 50 °C, and (c) RSF heated at 100 °C. Following thermal treatment, all samples were incubated with 10 µM Thioflavin T (ThT) for 24 h prior to imaging. Representative fluorescence images were acquired in the DAPI (intrinsic autofluorescence), FITC (ThT-associated fluorescence), and TRITC channels to qualitatively visualize temperature-dependent changes in aggregate morphology and fluorescence intensity. All images were acquired with a 50 µm scale bar.

AFM further supported this morphological progression at the nanoscale (Fig. 7a–f). Untreated RSF displayed heterogeneous surface features across the 10 × 10 µm scan area, while thermal treatment altered the distribution and prominence of surface protrusions. The average roughness (*R*_a_) changed only marginally from 16.40 nm for untreated RSF to 16.43 nm at 50 °C but increased to 19.48 nm at 100 °C; similarly, RMS roughness (*R*_q_) increased from 23.27 to 24.61 and 27.32 nm, respectively (Table S6 and Fig. S15–S17). Thus, the pronounced increase in surface-height variation at 100 °C is consistent with the greater morphological heterogeneity observed by SEM and fluorescence microscopy. This temperature-associated nanoscale reorganization is qualitatively consistent with Zhong *et al.*,^4^ while comparable increases in surface heterogeneity have been reported for thermally treated HSA/BSA and chemically modified hemoglobin.^14,41–43^ Overall, the findings demonstrate that thermal treatment of RSF is not a simple process of structural stabilization or aggregation, but a dynamic reorganization of the protein landscape in which changes at the molecular level are progressively translated into altered secondary structure, molecular ordering, and higher-order assembly. The ability of intrinsic aromatic-residue fluorescence to sensitively track this remodeling, together with the structural and morphological evidence obtained across multiple length scales, highlights the potential of RSF as a thermally tunable protein material whose organization can be followed through label-free optical signatures. More broadly, this work suggests that combining intrinsic protein fluorescence with complementary structural and morphological analyses can provide a powerful framework for linking subtle molecular changes to macroscopic material organization. This perspective opens opportunities to explore thermal processing not merely as a post-treatment step, but as a means of deliberately programming the structural landscape and functional properties of silk-based biomaterials.

**Fig. 7:**
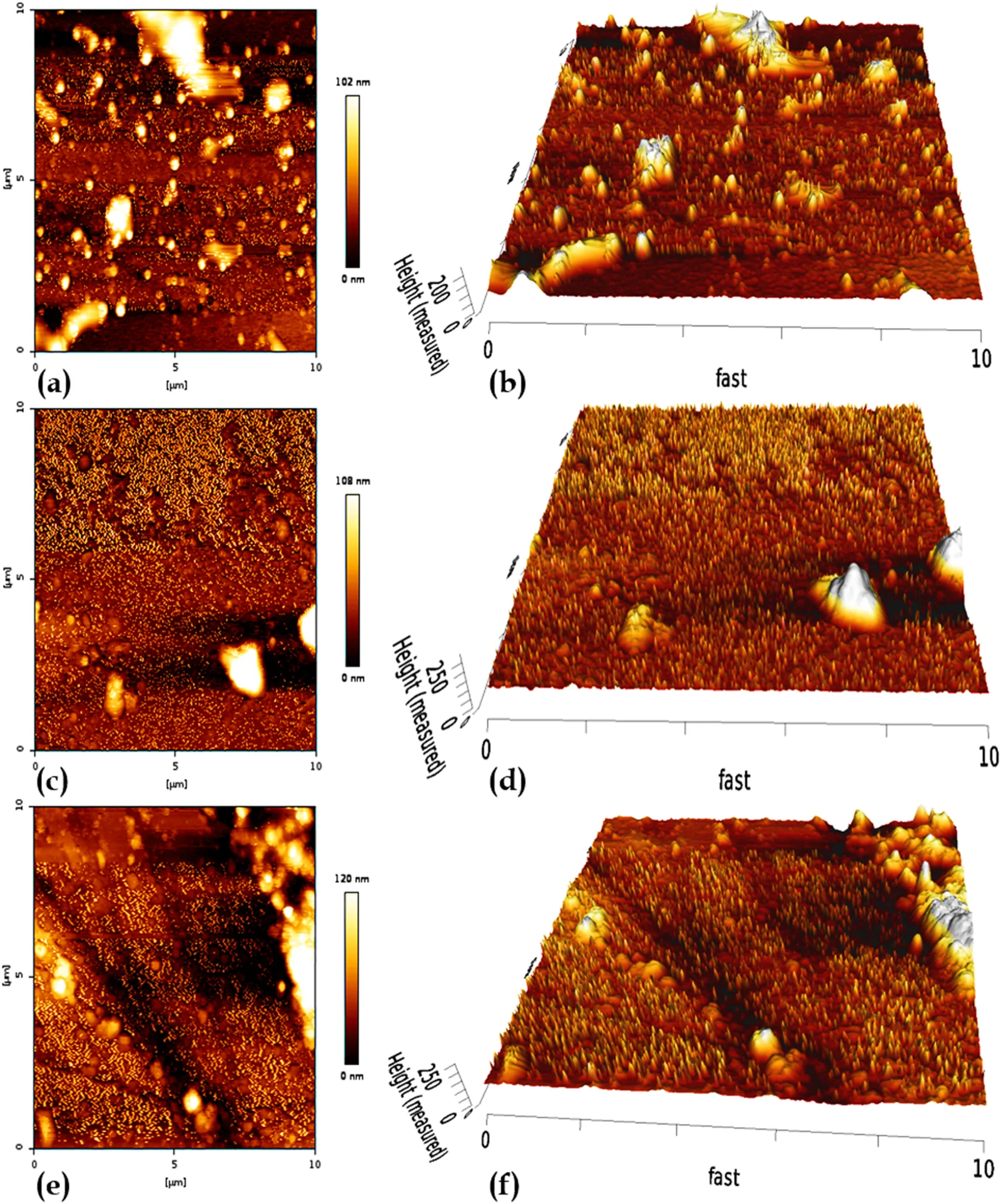
Atomic force microscopy (AFM) topographical characterization of untreated and thermally modified RSF. (a and b) Untreated RSF, showing the corresponding 2D height topography and 3D height representation; (c and d) RSF treated at 50 °C, showing the corresponding 2D height topography and 3D height representation; and (e and f) RSF treated at 100 °C, showing the corresponding 2D height topography and 3D height representation. The AFM images were acquired over a 10 × 10 µm scan area, with height variations represented by the corresponding color scales.

## 4. Conclusion, limitations and outlook

This study provided a multimodal evaluation of temperature-associated remodeling in RSF. Thermal treatment produced coordinated changes across several structural scales, including altered intrinsic fluorescence and fluorescence lifetime, increased accessibility of hydrophobic and dye-binding sites, redistribution of secondary-structure components, increased relative molecular ordering in the lyophilized state, and formation of larger and more heterogeneous assemblies. Together, these findings show that heating modifies the molecular environment and higher-order organization of RSF while retaining an overall β-sheet-rich structural framework. The combined measurements provided a more integrated view of RSF thermal remodeling than any single analytical method alone.

The present study has several limitations. Intrinsic fluorescence was evaluated across the full 50–100 °C range; however, the complementary structural, morphological, and dye-binding analyses were restricted to untreated RSF and representative samples treated at 50 and 100 °C. Therefore, the data establish temperature-associated differences between the selected conditions but do not define a continuous structural transition pathway at every intermediate temperature. In addition, the fluorescence, dye-binding, CD, FTIR, XRPD, DLS, SEM, and AFM measurements provide complementary evidence of structural reorganization, but they do not directly resolve the precise intermolecular interactions, aggregate architecture, hydration changes, hydrogen-bond rearrangements, or kinetic nucleation mechanisms responsible for the observed behavior. Likewise, the XRPD analysis supports increased relative crystalline ordering but does not demonstrate a definitive Silk I-to-Silk II crystalline phase transition.

Future studies should combine intermediate-temperature sampling with time-resolved structural measurements to distinguish temperature-dependent from time-dependent effects and to define the kinetics of RSF remodeling more precisely. Complementary approaches such as temperature-controlled CD or FTIR, fluorescence anisotropy, small- and wide-angle X-ray scattering, Raman spectroscopy, solid-state NMR, cryogenic electron microscopy, and advanced scattering-corrected fluorescence analysis could help resolve changes in molecular packing, hydration, intermolecular contacts, and aggregate architecture. Extending the work to RSF formulations prepared at different concentrations, pH values, ionic strengths, and processing histories would further clarify the generality of the observed thermal response and support the rational use of heat treatment to tailor silk-based materials for specific biomaterial applications.

## Author contributions

Shaik Basha, Darshan Chikkanayakanahalli Mukunda and Ameera K: writing – review & editing, writing – original draft, visualization, validation, software, project administration, methodology, investigation, formal analysis, data curation, conceptualization. Ahana Chattopadhyay: methodology, writing – review & editing. Krishna Kishore Mahato: writing – review & editing, visualization, validation, supervision, software, resources, project administration, investigation, funding acquisition, formal analysis, data curation, conceptualization.

## Conflicts of interest

The authors declare no competing financial interests.

## Supplementary Material

RA-016-D6RA06368C-s001

## Data Availability

The data supporting the findings of this study are available within the article and its supplementary information (SI). Additional datasets generated and/or analysed during the current study are available from the corresponding author upon reasonable request. Supplementary information: materials and methods, raw data files and related results. See DOI: https://doi.org/10.1039/d6ra06368c.
