# Supplementary material for: Multimodal characterization of temperature-induced structural remodeling and aggregation of regenerated silk fibroin
Source: RSC Adv. 2026 Oct 2;16(46):59762–75. doi: 10.1039/d6ra06368c (PMC13632631; doi:10.1039/d6ra06368c)
Supplement: Supplementary file 1 [file RA-016-D6RA06368C-s001.pdf]

Supplementary Information (SI) for RSC Advances.
This journal is © The Royal Society of Chemistry 2026
Supplementary Information (SI) for RSC Advances.
This journal is © The Royal Society of Chemistry

Multimodal Characterization of Temperature-Induced Structural Remodeling and Aggregation of Regenerated Silk Fibroin

Shaik Basha [1,†], Darshan Chikkanayakanahalli Mukunda[1,2,†], Ameera K[1,†], Ahana Chattopadhyay[1], Krishna Kishore Mahato[1,]*

1. *Department of Biophysics, Manipal School of Life Sciences, Manipal Academy of Higher Education, Manipal, India.*
2. *Department of Biosciences & Bioengineering, Indian Institute of Technology Dharwad, Dharwad - 580011, Karnataka, India.*

† Authors with equal contribution

****Corresponding author:*** *mahato.kk@manipal.edu & kkmahato@gmail.com*

1. Materials and Methods

1.1. Fluorescence Spectroscopy Study

Intrinsic fluorescence spectroscopy was employed to monitor temperature-induced microenvironment changes of endogenous fluorophores (e.g., tyrosine) of untreated and thermally modified regenerated silk fibroin (RSF). Fluorescence measurements were performed using a light-emitting diode–induced autofluorescence (LED-IAF) system with an excitation wavelength of 285 nm, selectively exciting aromatic amino acid residues, primarily tryptophan (Trp) and tyrosine (Tyr), which are sensitive to changes in local microenvironment during protein folding and aggregation processes. Emission spectra were recorded over the range of 300–600 nm using a high-resolution spectrofluorometer (QE Pro, Ocean Optics Inc., USA) to ensure adequate spectral sensitivity and resolution. All fluorescence measurements were conducted at room temperature (20 ± 1 °C) to eliminate thermal artifacts during spectral acquisition. Detailed descriptions of the instrumental configuration, calibration, and measurement protocol have been reported in our previous study [1]. Based on the outcomes of the intrinsic fluorescence analysis, representative RSF samples, including untreated RSF, RSF incubated at 50 °C, and RSF incubated at 100 °C, were selected for subsequent structural, spectroscopic, and microscopic investigations. These conditions were selected to capture minimally perturbed and highly thermally modified states and to evaluate associated changes in secondary structure, molecular ordering, aggregation, and morphology without extrapolating beyond the experimental observations.

1.2. UV-Visible Absorption Spectroscopy

Ultraviolet–visible (UV–Vis) absorption spectroscopy was employed to examine temperature-induced structural changes and aggregation behavior of untreated RSF and thermally modified RSF samples incubated at 50 °C and 100 °C. Absorption spectra were recorded using a Tecan Spark multimode microplate reader (Männedorf, Switzerland) over a wavelength range of 220–600 nm. Protein solutions were dispensed into UV-transparent, flat-bottom 96-well microplates, with 200 µl of RSF solution added per well. Particular attention was given to absorbance at 280 nm, which arises predominantly from aromatic amino acid residues, namely tryptophan (Trp) and tyrosine (Tyr), and is commonly used to assess changes associated with protein conformational state. All measurements were performed in triplicate (N = 3) to ensure reproducibility and data reliability. The protocol was adapted from our previous study [1].

1.3. Lifetime measurement

Time-correlated single-photon counting (TCSPC) fluorescence lifetime measurements were performed to investigate changes in the excited-state dynamics of intrinsic fluorophores in untreated and thermally modified RSF. Measurements were carried out using a HORIBA Fluorolog-QM™ Series photoluminescence spectrometer equipped with lifetime measurement capability. RSF samples at a concentration of 0.2 mg/ml, including untreated RSF and samples thermally treated at 50 and 100 °C, were analyzed at room temperature under identical measurement conditions. The samples were excited at 280 nm, and fluorescence decay was monitored at the emission maximum of 320 nm. The decay profiles were analyzed using a biexponential decay model, and the corresponding lifetime parameters were obtained from the fitted decay curves. The quality of the fits was evaluated using the reduced chi-square (χ^2) value together with residual and autocorrelation analyses. The obtained lifetime parameters were used for comparative evaluation of changes in the fluorescence dynamics of RSF following thermal treatment.

1.4. Thioflavin T Assay

The Thioflavin T (ThT) fluorescence assay was employed to assess the formation of β-sheet-rich structures in untreated RSF and thermally modified RSF samples incubated at 50 °C and 100 °C. A ThT stock solution (1 mM) was prepared in Milli-Q water, filtered to remove particulates, and diluted to a final working concentration of 10 µM immediately prior to use.

For each measurement, 100 µl of untreated or thermally modified RSF solution was mixed with 900 µl of 10 µM ThT solution, following a protocol adapted from our previous study [1]. The resulting mixtures were transferred to UV-transparent, flat-bottom 96-well microplates, with 200 µl loaded per well. Fluorescence measurements were performed using a Tecan Spark multimode microplate reader (Männedorf, Switzerland) with an excitation wavelength of 400 nm, and emission spectra were recorded over the range of 450–700 nm. All measurements were conducted in triplicate (N = 3) to ensure reproducibility. For the highest temperature condition (100 °C), additional time-resolved fluorescence measurements were performed to probe aggregation kinetics. During incubation, aliquots were withdrawn at 30 min intervals and analyzed using the ThT assay to monitor the temporal evolution of β-sheet-rich structural organization.

1.5. ANS Assay

The 8-anilino-1-naphthalenesulfonic acid (ANS) fluorescence assay was employed to assess changes in surface hydrophobicity of untreated RSF and thermally modified RSF samples incubated at 50 °C and 100 °C. A stock solution of ANS (500 µM) was prepared in Milli-Q water. For each measurement, 100 µl of untreated or thermally modified RSF solution was mixed with 500 µl of the ANS stock solution, following a protocol adapted from our previous study [1]. The resulting mixtures were transferred to flat-bottom 96-well microplates, with 200 µl loaded per well. Fluorescence measurements were performed using a Tecan Spark multimode microplate reader (Männedorf, Switzerland) with an excitation wavelength of 350 nm, and emission spectra were recorded over the range of 400–800 nm. All measurements were conducted in triplicate (N = 3) to ensure data reproducibility. For the highest temperature condition (100 °C), additional time-resolved fluorescence measurements were performed to probe aggregation kinetics. During incubation, aliquots were withdrawn at 30 min intervals and analyzed using the ANS assay to monitor temporal changes in hydrophobic surface exposure.

1.6. Congo Red Assay

To further assess temperature-dependent structural organization in RSF, a Congo Red (CR) binding assay was performed following a protocol adapted from our previous study [1]. A CR stock solution (1 mM) was prepared in Milli-Q water and diluted to a final working concentration of 20 µM for all experiments. For each measurement, 100 µl of untreated or

thermally modified RSF solution was mixed with 500 µl of the CR solution (20 µM) and allowed to equilibrate at room temperature for 2–3 min. The resulting mixtures were transferred to UV-transparent, flat-bottom 96-well microplates, and absorbance spectra were recorded over the wavelength range of 300–600 nm using a Tecan Spark multimode microplate reader (Männedorf, Switzerland). All measurements were performed in triplicate (N = 3) to ensure data reproducibility. For the highest temperature condition (100 °C), additional time-resolved measurements were conducted to probe aggregation kinetics. During incubation, aliquots were withdrawn at 30 min intervals and analyzed using the CR assay to monitor temporal changes in dye binding behavior.

1.7. Fourier Transform Infrared Spectroscopy (FTIR)

FTIR spectroscopy was employed to assess untreated and thermally modified RSF. The lyophilized samples were recorded using a single-reflection diamond attenuated total reflectance (ATR) accessory on an FT/IR-4X spectrometer (JASCO Ltd., Tokyo, Japan) equipped with a mercury cadmium telluride (MCT) detector, which was used to record the spectra from lyophilized protein samples over a spectral range of 4000–600 cm^{-1} to capture characteristic vibrational modes associated with protein secondary structures. Background spectra were subtracted to enhance the signal-to-noise ratio. Further, the transmittance FTIR spectra of untreated and thermally modified RSF were converted to absorbance using equation no. 1, where A is absorbance, and T is transmittance (%). Further, a region of interest (1580 – 1720 cm^{-1}) was selected and subsequently smoothened (to reduce the noise) using the Savitzky–Golay algorithm, followed by unity-based normalization and baseline correction using Spectragryph software (version 1.2.16.1). Secondary-structure quantification was performed as per previous reported studies [1] exclusively using Amide - I (1600–1700 cm^{-1}) band deconvolution (Gaussian curve fitting using OriginPro 2026).

$$A = -\log_{10}\left(\frac{T}{100}\right)$$ ………… **Eq. (1)**

The percentage secondary-structure contribution of each secondary structural component was calculated using equation no. 2.

Secondary structure content $$\%_i = \frac{A_i}{\Sigma A} \times 100$$ ………… **Eq. (2)**

Where A_i represents the integrated area of the ith Gaussian peak obtained from Amide I band deconvolution, and ΣA is the sum of areas of all fitted amide I components.

1.8. Circular Dichroism Study

Circular dichroism (CD) spectroscopy was performed to evaluate changes in the secondary structural characteristics of untreated RSF and thermally modified RSF samples. Far-UV CD spectra were recorded over the wavelength range of 190–260 nm using a J-810 spectropolarimeter (JASCO, Japan) equipped with a 1 mm path-length quartz cuvette. Spectra were acquired at room temperature and represent the average of three consecutive scans after subtraction of the corresponding buffer baseline. The observed ellipticity values (θobs, mdeg) were converted to mean residue ellipticity (MRE) using equation no 3.

$$\text{MRE } (\text{deg}\cdot\text{cm}^2\cdot\text{dmol}^{-1}) = \theta\text{obs (mdeg)} / [10 \times C \times n \times l] \;\ldots\ldots\ldots\ldots \textbf{Eq. (3)}$$

where C is the molar concentration of RSF, n is the number of amino acid residues in the regenerated silk fibroin (885 residues), and l is the optical path length of the cuvette (0.1 cm). Secondary structural components were estimated by full-spectrum deconvolution of MRE spectra using the BeStSel algorithm, and the resulting parameters were used for comparative analysis between untreated RSF and thermally modified RSF samples [1].

1.9. Scanning Electron Microscopy

The surface morphology of untreated RSF and thermally modified RSF lyophilized samples was examined using a JEOL IT300LV scanning electron microscope (JEOL Ltd., Tokyo, Japan). All measurements were performed at room temperature (25 °C). Prior to imaging, lyophilized samples were mounted on aluminum stubs using carbon adhesive tape and sputter-coated with a thin layer of gold to enhance surface conductivity and image quality. Scanning electron micrographs were acquired at multiple magnifications to visualize morphological features and assess differences between untreated and thermally modified RSF samples [1].

1.10. Fluorescence Microscopy

Fluorescence microscopy was employed to visualize aggregation behavior and structural organization in untreated RSF and thermally modified RSF samples. For this analysis, 1 ml of each RSF solution was incubated with ThT at a final concentration of 10 µM for 24 h at room temperature. Following incubation, a 100 µl aliquot of each sample was placed onto a clean

glass slide, gently covered with a coverslip, and immediately examined using an inverted fluorescence microscope (CKX53, Olympus, Japan). Samples were excited using ultraviolet (UV) illumination to detect intrinsic protein fluorescence arising primarily from aromatic amino acid residues, including tryptophan (Trp) and tyrosine (Tyr), as well as ThT-associated fluorescence. Fluorescence images were acquired using identical acquisition settings for all samples. A scale bar of 50 µm was applied to all images to facilitate comparative assessment of aggregate morphology and distribution under different experimental conditions [1].

1.11. Atomic Force Microscopy

Atomic Force Microscopy (AFM) was carried out using a NanoWizard® 3 AFM system (JPK Instruments, Germany) to visualize morphological changes in untreated RSF and thermally modified RSF samples. Protein samples were diluted 1:10 immediately before imaging. A 5 µl aliquot of each diluted sample was drop-cast onto a clean Muscovite Mica for AFM (AFM-71856-03) and allowed to adsorb for 2 min, followed by gentle drying under ambient conditions. AFM imaging was performed in tapping mode using silicon cantilevers under ambient air. Topography images were acquired at a scan size of 10 µm × 10 µm to capture aggregate-level surface features. For each protein, native (4 °C) samples were compared directly with those heated at 50 °C and 100 °C. Height maps, 3D rendered height traces, and roughness parameters were extracted using the JPK Data Processing software.

1.12. X-ray powder diffraction (XRPD) Analysis

XRPD patterns of untreated RSF and thermally modified RSF samples, both in their native state and after thermal treatment at 50°C and 100 °C of lyophilized proteins, were recorded using a Malvern PANalytical® Empyrean Series 3 X-ray diffractometer. Diffraction scans were collected over a 2θ range of 5°–50°, with a step size of 0.02° and a counting time of 1 s per step, enabling the detection of subtle changes in structural ordering and aggregation induced by heat treatment. The spectral deconvolution of the XRPD profiles was performed using the OriginPro 2026. The diffraction profiles were fitted using the Gaussian peak functions to resolve the individual crystalline reflections. The deconvolution procedure enabled the separation and quantification of the crystalline and amorphous components based on their integrated peak areas. The crystallinity index (CI) was calculated from the ratio of the integrated crystalline area to the total integrated area of the XRPD profile [2] .

1.13. Dynamic Light Scattering Analysis

Dynamic light scattering (DLS) measurements were performed to determine the hydrodynamic size distribution of protein assemblies in untreated RSF and thermally modified RSF samples. Measurements were carried out using a Zetasizer Nano ZS90 instrument (Malvern Instruments Ltd., UK). The refractive index of the protein was set to 1.45, while the refractive index of the dispersant (Milli-Q water) was set to 1.33, with an absorption (extinction) coefficient of 0.960. For each sample, measurements were performed in triplicate, with each run consisting of 12 consecutive measurements. The intensity-weighted Z-average hydrodynamic diameter and polydispersity index (PDI) were recorded for each measurement. The Z-average hydrodynamic diameter was used as the representative measure of particle size for comparison among the treatment groups.

2. Results

2.1. Fluorescence Lifetime Measurements

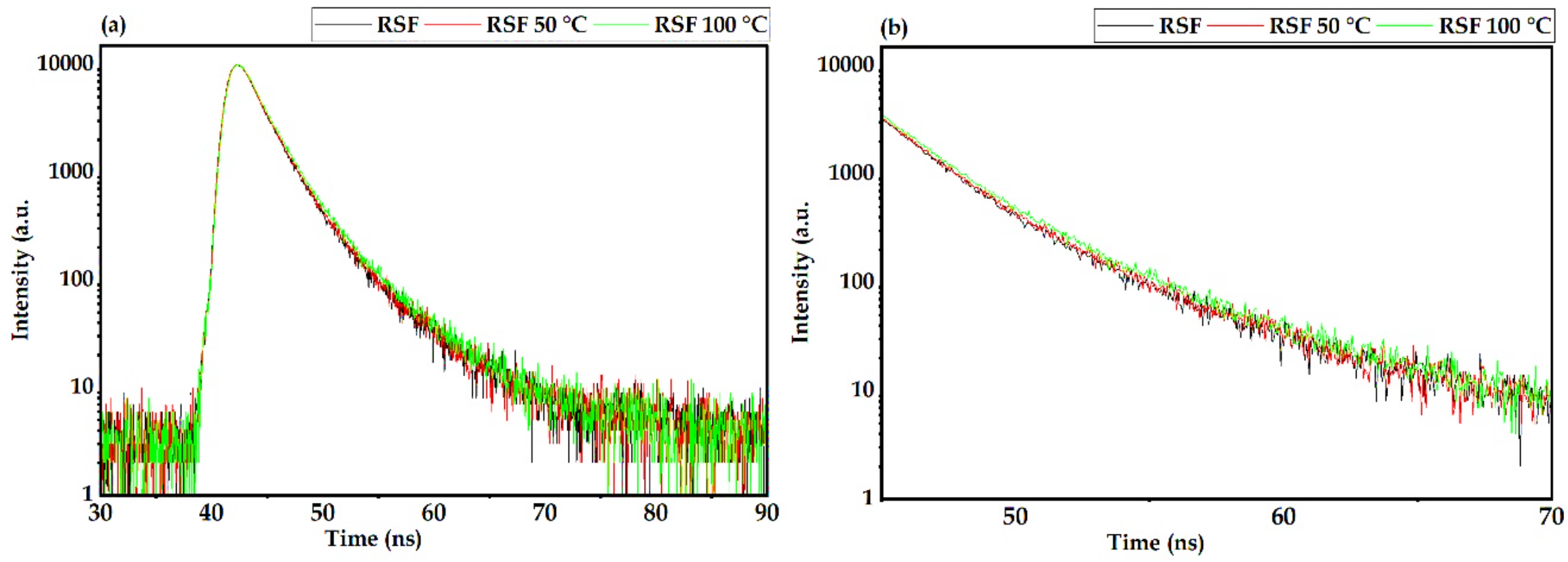

Figure S1. TCSPC fluorescence decay profiles of untreated RSF and thermally treated RSF at 50 and 100 °C, recorded at 280 nm excitation and 320 nm emission. (a) Full decay profiles and (b) expanded view of the decay region.

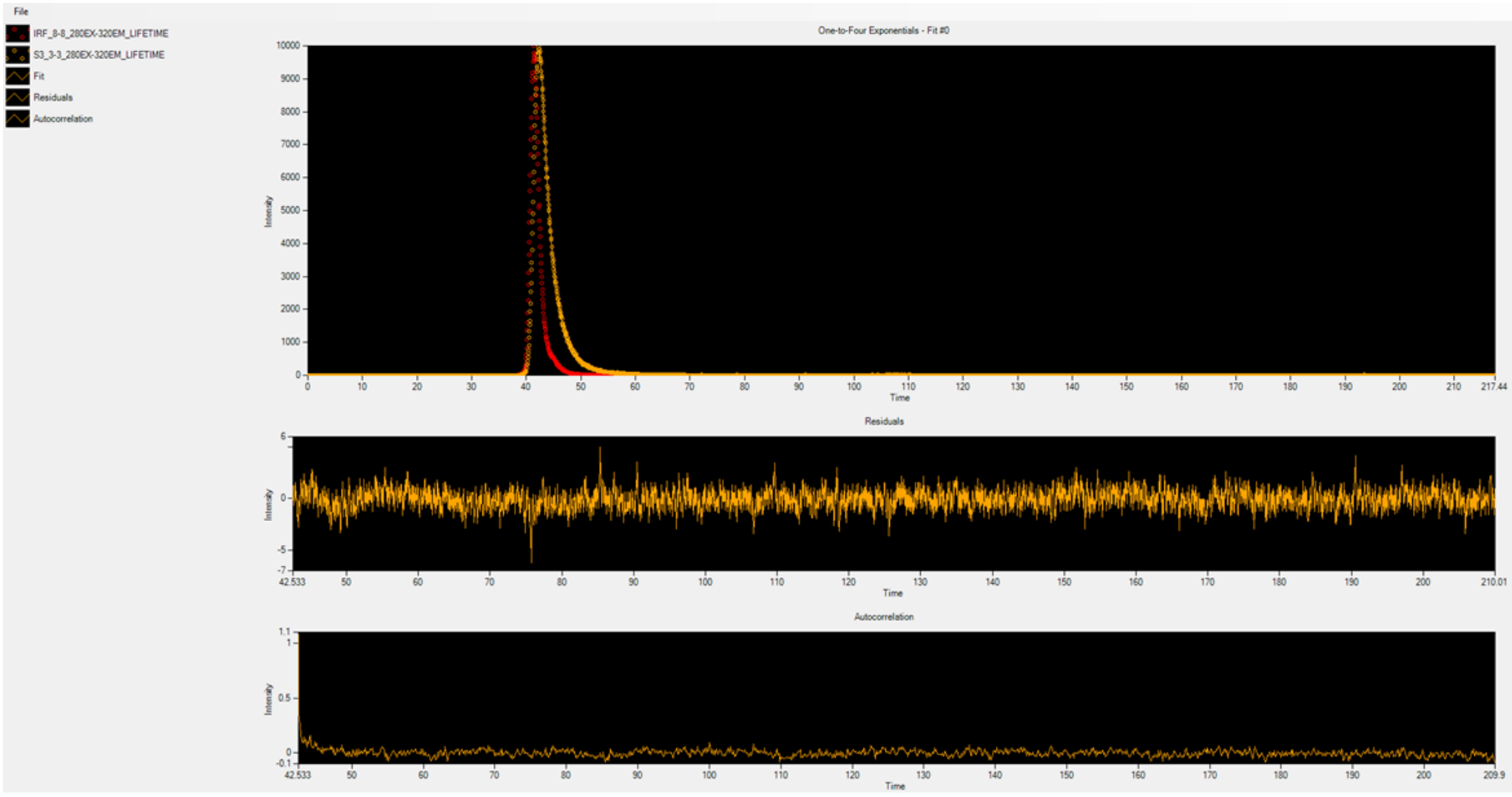

Figure S2. TCSPC fluorescence decay fitting of untreated RSF at 280 nm excitation and 320 nm emission.

```
One-To-Four Exponentials
------------------------

Fit # 0

2026-09-03 11:51:58.074

Trace list:
-----------
IRF:             IRF_8-8_280EX-320EM_LIFETIME
Decay:           S3_3-3_280EX-320EM_LIFETIME

Initial Parameter Summary:
--------------------------
Lifetimes:       2
Start Time:      4.25300E+001
End Time:        2.10060E+002
tau[1]=          1.51334E+000      fixed=no      auto=yes
tau[2]=          4.54003E+000      fixed=no      auto=yes
a[0]=            1.00000E+000
a[1]=            1.00000E+000
Shift=           0.00000E+000      fixed=no
Offset=          0.00000E+000      fixed=no
X Offset Mode:   None
X Offset:        0.00000E+000
SPC Data:        no
-------------------------------------------------------------
Iterations:      11
Elapsed time:    00:00:02.752

Chi2:            1.12916E+000
Durbin Watson:   1.43574E+000
Z:               -2.38727E-003

tau[1]:          1.30869E+000 ± 7.18151E-003
tau[2]:          4.51702E+000 ± 3.87019E-002
a[1]:            1.13821E+000 ± 5.05348E-003 (9.42944E+001 ± 4.18653E-001%)
a[2]:            6.88711E-002 ± 1.71290E-003 (5.70559E+000 ± 1.41904E-001%)

Ampl aver tau:   1.49175E+000
Inten aver tau:  1.86298E+000

Integ Int.[1]:   8.27235E-001
Integ Int.[2]:   1.72765E-001

Offset:          -2.30408E-001
Shift:           -1.39533E-002
-------------------------------------------------------------
Done.
```

Figure S3. TCSPC fluorescence decay fit, residuals, and autocorrelation analysis of untreated RSF using a biexponential model.

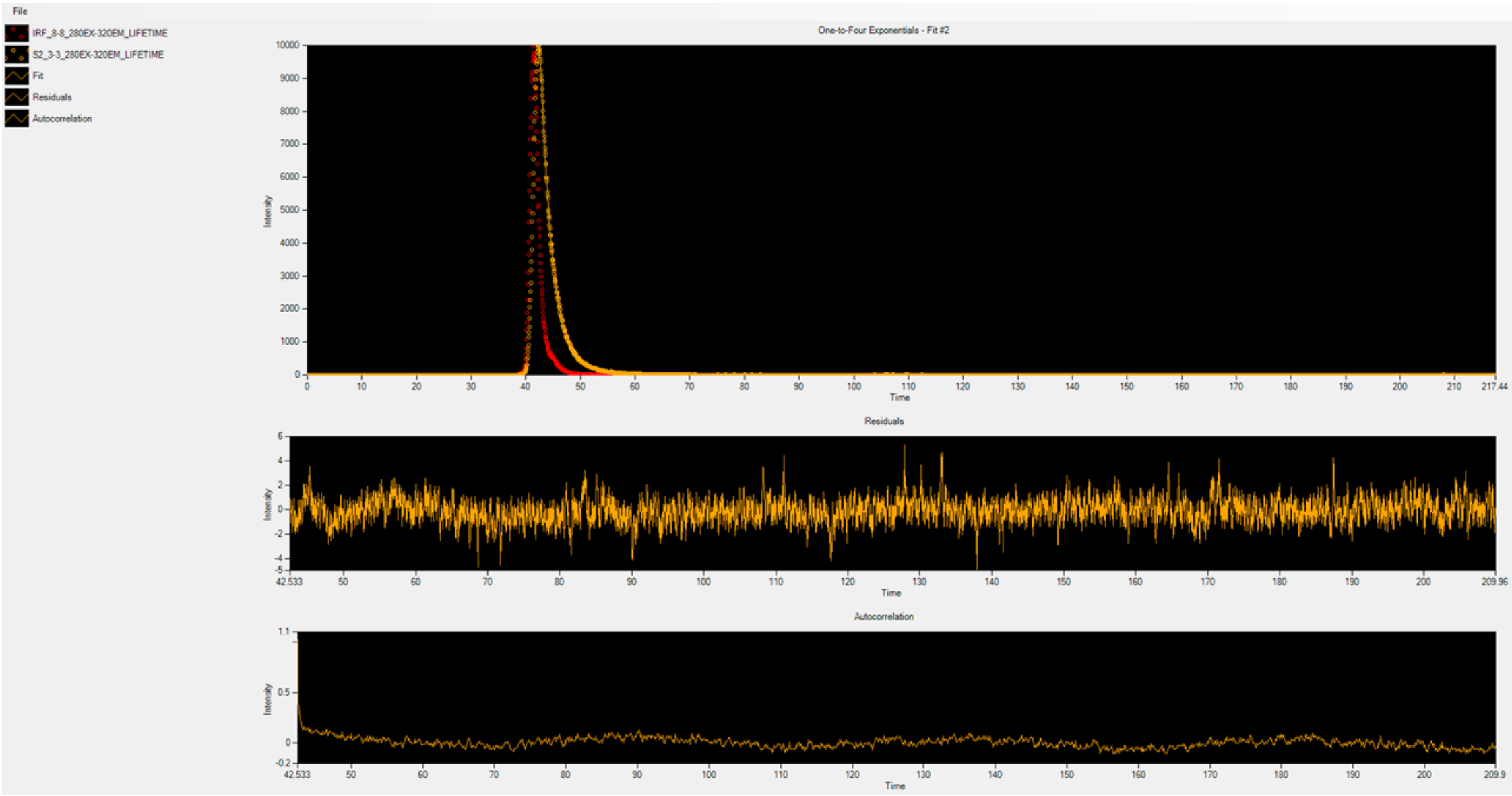

Figure S4. TCSPC fluorescence decay fitting of RSF treated at 50 °C at 280 nm excitation and 320 nm emission.

```
One-To-Four Exponentials
------------------------

Fit # 2

2026-09-03 11:49:12.610

Trace list:
-----------
IRF:             IRF_8-8_280EX-320EM_LIFETIME
Decay:           S2_3-3_280EX-320EM_LIFETIME

Initial Parameter Summary:
--------------------------
Lifetimes:       2
Start Time:      4.25300E+001
End Time:        2.10000E+002
tau[1]=          1.46024E+000       fixed=no      auto=yes
tau[2]=          4.38072E+000       fixed=no      auto=yes
a[0]=            1.00000E+000
a[1]=            1.00000E+000
Shift=           0.00000E+000       fixed=no
Offset=          0.00000E+000       fixed=no
X Offset Mode:   None
X Offset:        0.00000E+000
SPC Data:        no
-------------------------------------------------------------
Iterations:      9
Elapsed time:    00:00:02.80

Chi2:            1.30708E+000
Durbin Watson:   1.71906E+000
Z:               -1.02386E-001

tau[1]:          1.34890E+000 ± 7.74916E-003
tau[2]:          4.48690E+000 ± 3.52490E-002
a[1]:            1.15102E+000 ± 6.94285E-003 (9.37424E+001 ± 5.65445E-001%)
a[2]:            7.68346E-002 ± 1.87590E-003 (6.25763E+000 ± 1.52779E-001%)

Ampl aver tau:   1.54526E+000
Inten aver tau:  1.91907E+000

Integ Int.[1]:   8.18300E-001
Integ Int.[2]:   1.81700E-001

Offset:          -6.79972E-001
Shift:           1.19838E-001
-------------------------------------------------------------
Done.
```

Figure S5. TCSPC fluorescence decay fit, residuals, and autocorrelation analysis of RSF treated at 50 °C using a biexponential model.

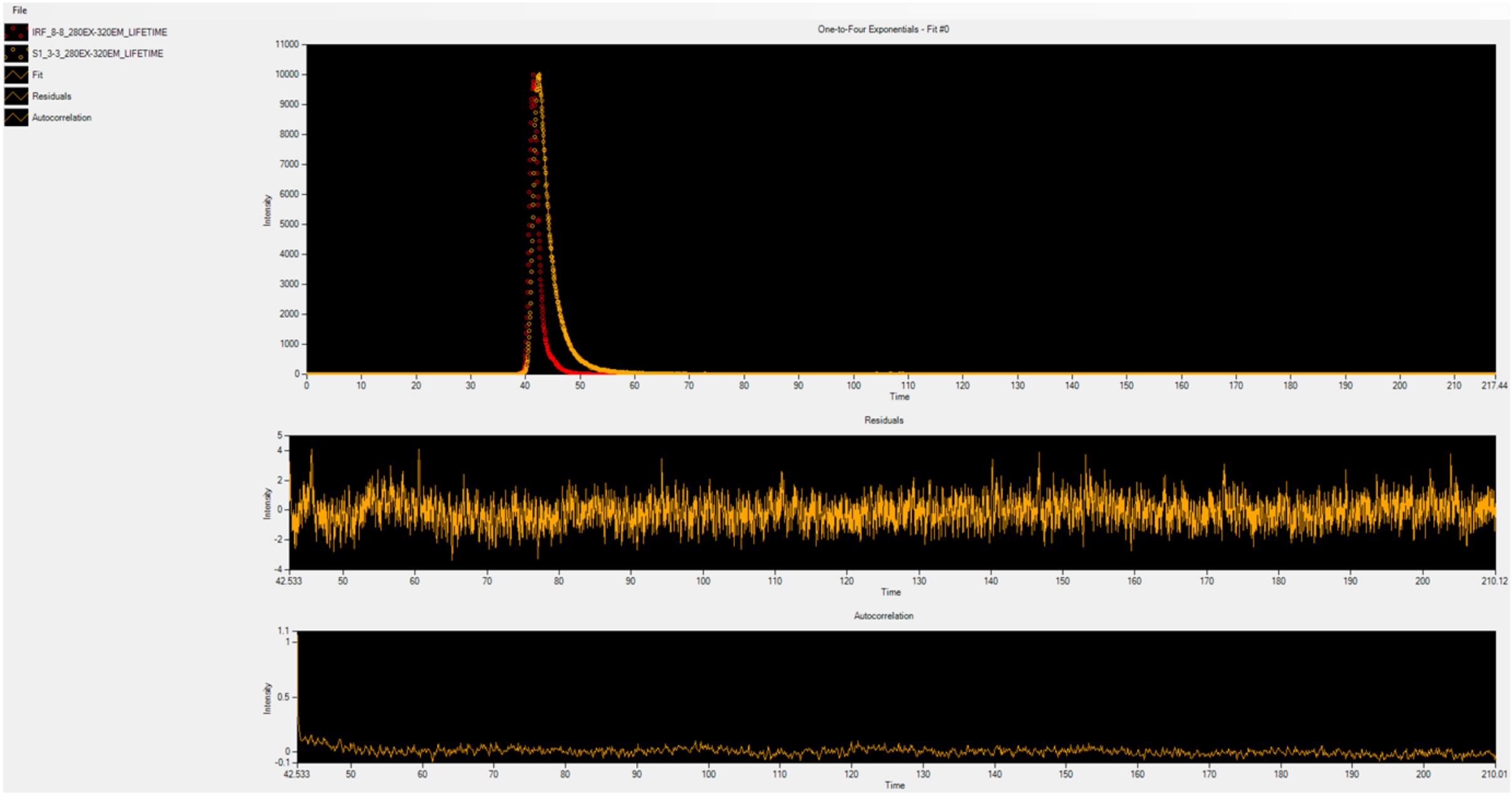

Figure S6. TCSPC fluorescence decay fitting of RSF treated at 100 °C at 280 nm excitation and 320 nm emission.

```
One-To-Four Exponentials
------------------------

Fit # 0

2026-09-03 11:41:19.682

Trace list:
-----------
IRF:                IRF_8-8_280EX-320EM_LIFETIME
Decay:              S1_3-3_280EX-320EM_LIFETIME

Initial Parameter Summary:
--------------------------
Lifetimes:          2
Start Time:         4.25300E+001
End Time:           2.10120E+002
tau[1]=             1.53989E+000        fixed=no        auto=yes
tau[2]=             4.61967E+000        fixed=no        auto=yes
a[0]=               1.00000E+000
a[1]=               1.00000E+000
Shift=              0.00000E+000        fixed=no
Offset=             0.00000E+000        fixed=no
X Offset Mode:      None
X Offset:           0.00000E+000
SPC Data:           no
-------------------------------------------------------------
Iterations:         13
Elapsed time:       00:00:03.218

Chi2:               1.09973E+000
Durbin Watson:      1.24655E+000
Z:                  -6.74273E-002

tau[1]:             1.37702E+000 ± 7.80478E-003
tau[2]:             4.47622E+000 ± 3.05574E-002
a[1]:               1.12539E+000 ± 6.37365E-003 (9.27593E+001 ± 5.25342E-001%)
a[2]:               8.78471E-002 ± 1.82460E-003 (7.24071E+000 ± 1.50391E-001%)

Ampl aver tau:      1.60143E+000
Inten aver tau:     2.00426E+000

Integ Int.[1]:      7.97612E-001
Integ Int.[2]:      2.02388E-001

Offset:             -7.45267E-001
Shift:              6.93883E-002
-------------------------------------------------------------
Done.
```

Figure S7. TCSPC fluorescence decay fit, residuals, and autocorrelation analysis of RSF treated at 100 °C using a biexponential model.

Table S1. TCSPC fluorescence lifetime parameters of untreated and thermally modified RSF.

Sample	τ_1 (ns)	τ_2 (ns)	Amplitude-average lifetime (ns)	Intensity-average lifetime (ns)	χ^2
RSF	1.30869 ± 0.00718	4.51702 ± 0.03870	1.49175	1.86298	1.12916
RSF at 50 °C	1.34890 ± 0.00775	4.48690 ± 0.03525	1.54526	1.91907	1.30708
RSF at 100 °C	1.37702 ± 0.00780	4.47622 ± 0.03056	1.60143	2.00426	1.09973

Note: Fluorescence decay profiles were fitted using a biexponential model. Measurements were performed at 280 nm excitation and 320 nm emission using a HORIBA Fluorolog-QM™ fluorescence lifetime spectrometer. χ^2 represents the reduced chi-square value for the corresponding fit.

2.2. Fourier Transform Infrared Spectroscopy (FTIR)

Table S2. Secondary-structure composition of untreated and thermally modified RSF determined by FTIR Amide I band deconvolution.

Secondary structure	Untreated RSF (%)	RSF at 50 °C (%)	RSF at 100 °C (%)
α-helix	17.34	20.65	8.43
β-sheet	75.34	48.67	54.00
β-turn	3.23	23.35	24.29
Random coil	4.09	7.33	13.28

2.3. Circular Dichroism (CD) spectroscopy

Table S3. BeStSel-estimated secondary structure composition of RSF before and after thermal treatment.

Sample	Helix (%)	Antiparallel β-sheet (%)	Parallel β-sheet (%)	Turn (%)	Others (%)	RMSD	NRMSD

Untreated RSF	3.4	41.5	0.0	13.0	42.1	0.0537	0.01941
RSF, 50 °C	1.7	43.7	0.0	11.7	42.9	0.0866	0.02995
RSF, 100 °C	0.0	45.3	0.0	13.0	41.8	0.0391	0.04170

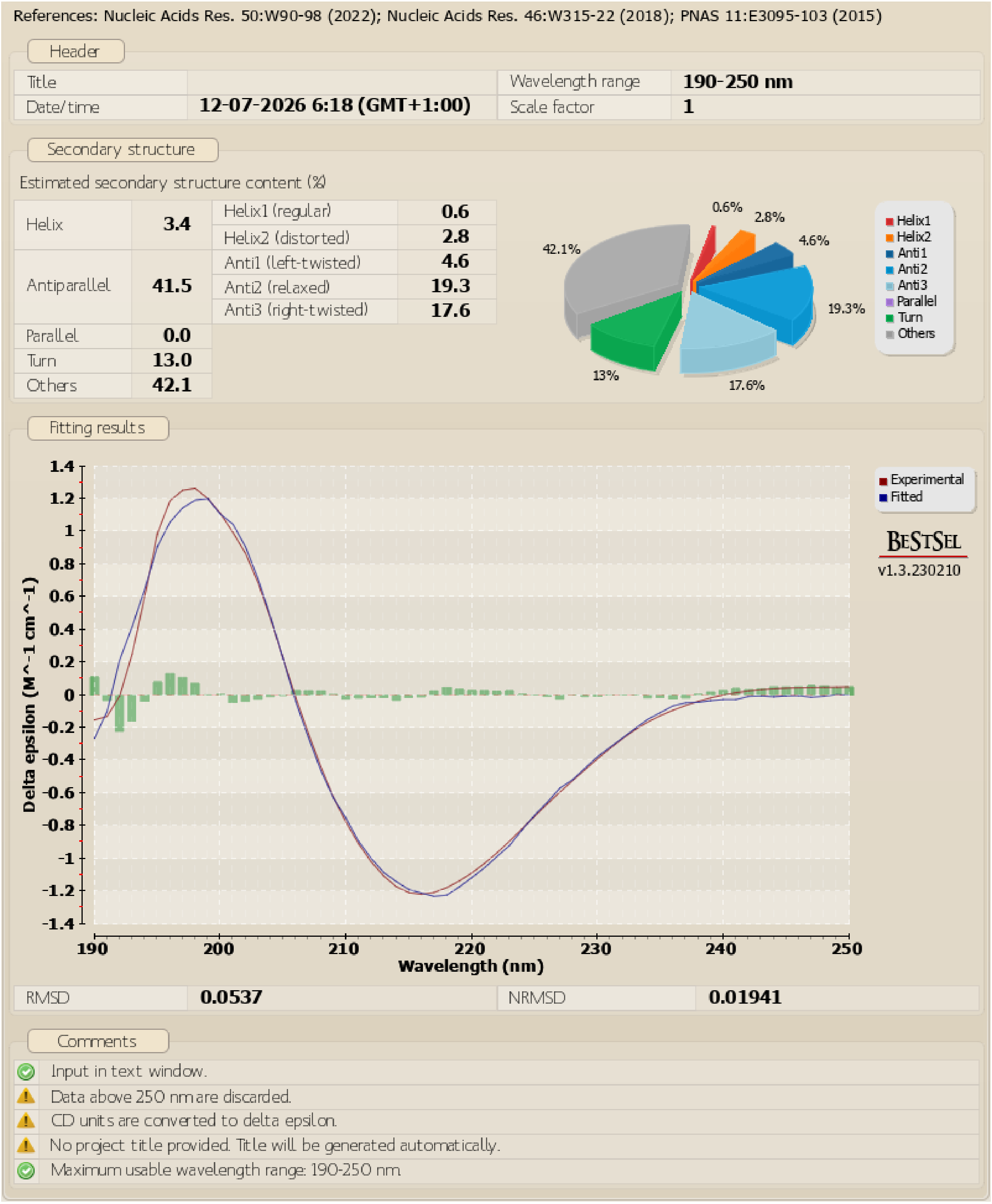

Figure S8. BeStSel secondary structure analysis of regenerated silk fibroin (RSF).

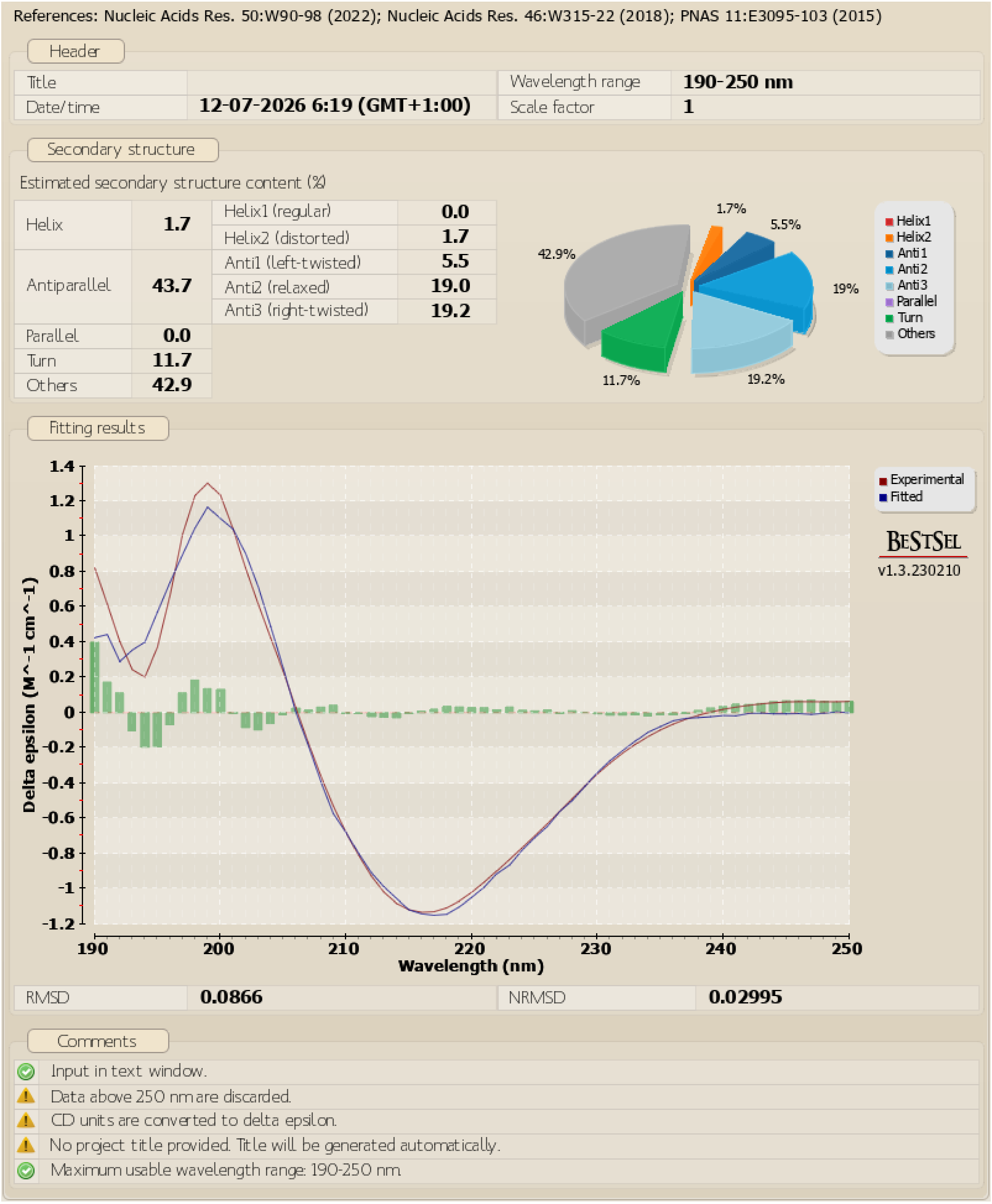

Figure S9. BeStSel secondary structure analysis of regenerated silk fibroin (RSF) thermally treated at 50 °C.

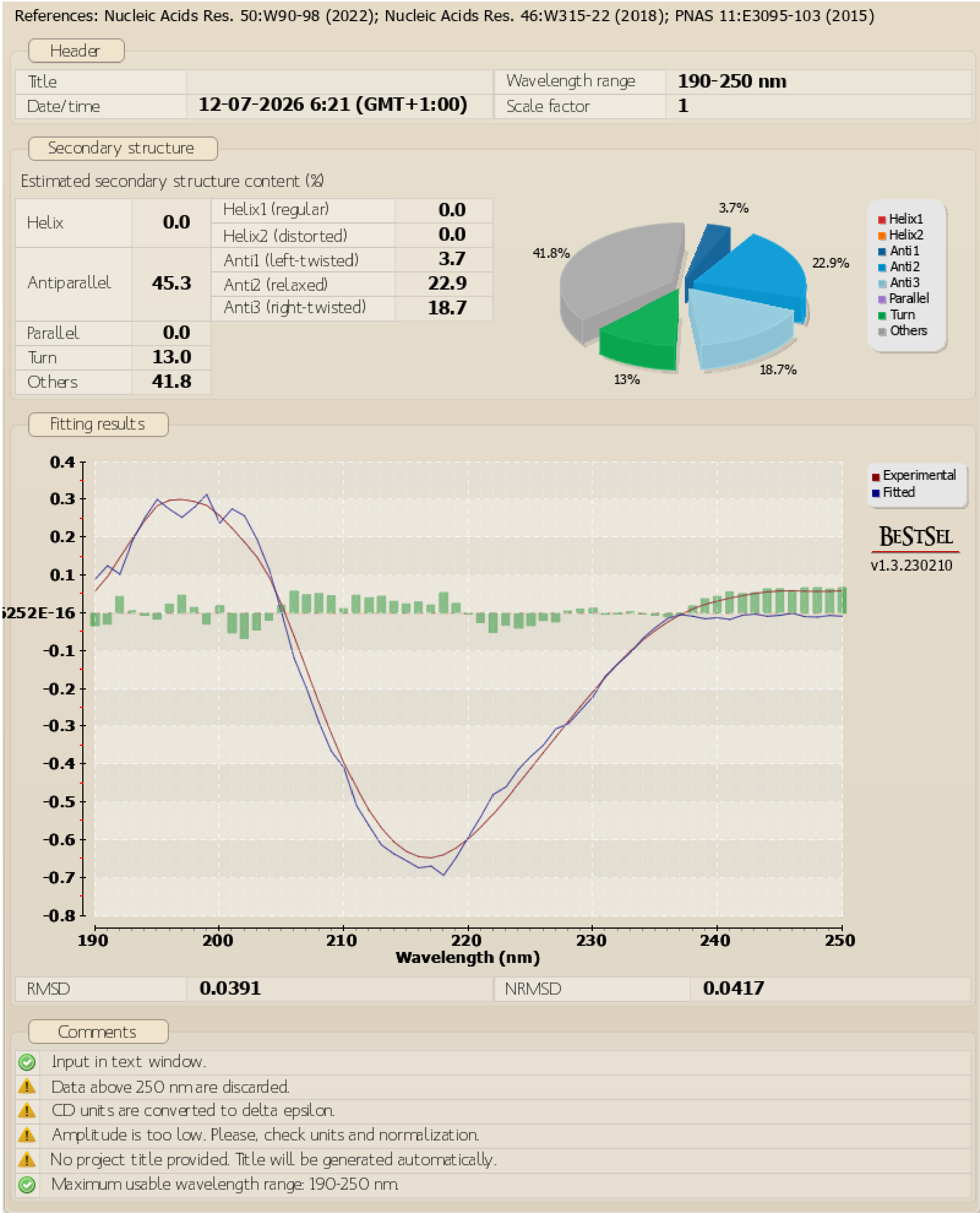

Figure S10. BeStSel secondary structure analysis of regenerated silk fibroin (RSF) thermally treated at 100 °C.

2.4. X-ray powder diffraction (XRPD) Analysis

Table S4. XRPD-derived total, crystalline, and amorphous areas, with corresponding crystallinity (Xc) and amorphous fraction of native and thermally treated RSF.

Sample	Total Area (A_{total})	Crystalline Area (A_c)	Amorphous Area (A_a)	Crystallinity (X_c)	Amorphous Fraction
**RSF**	6463.56665	2934.80489	3528.76176	45.41%	54.59%
**RSF at 50 ºC**	8026.22194	4200.12827	3826.09367	52.33%	47.67%
**RSF at 100 ºC**	9253.61664	6555.91760	2697.69904	70.85%	29.15%

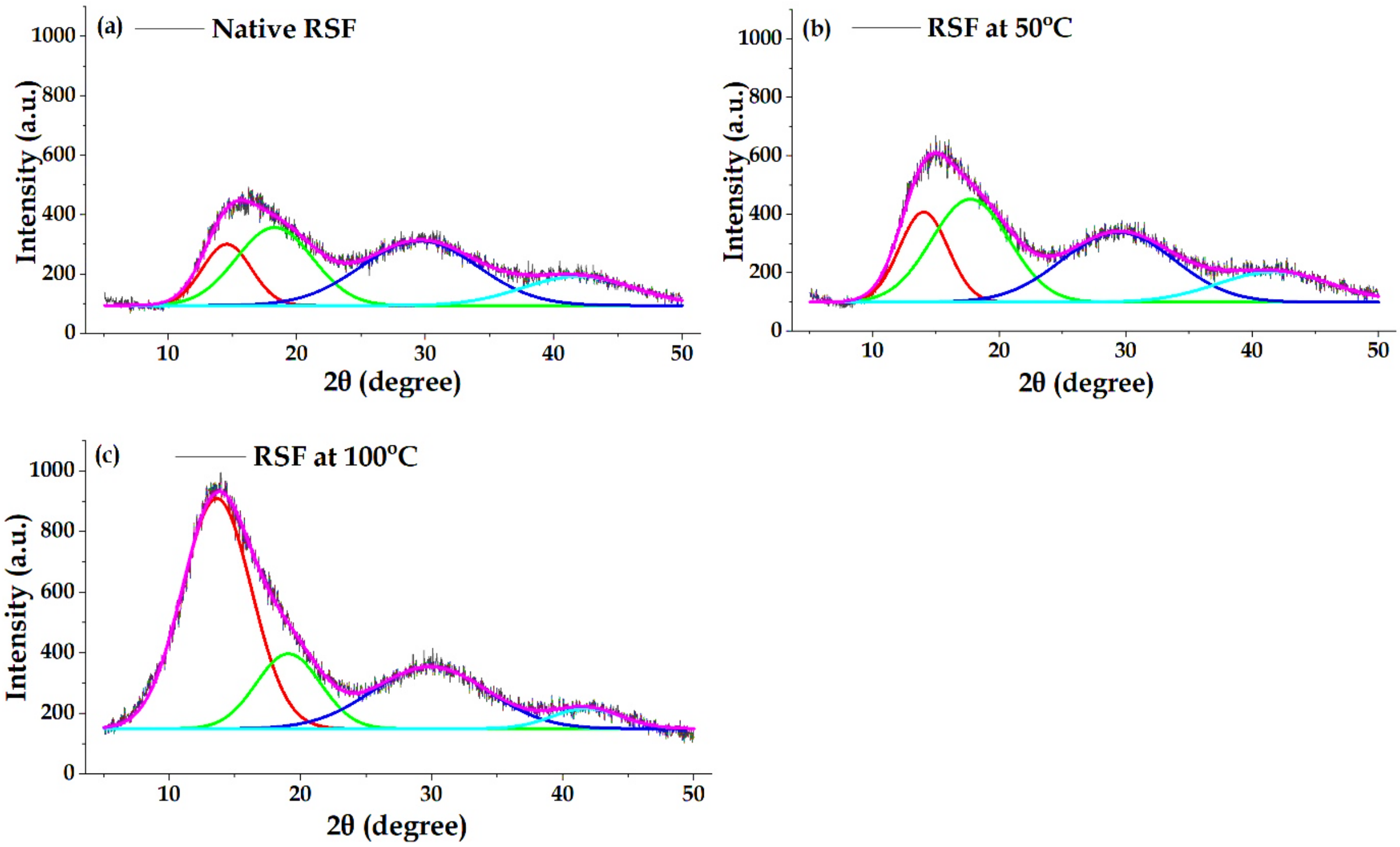

Figure S11. Deconvoluted X-ray powder diffraction (XRPD) patterns of (a) native RSF, (b) RSF treated at 50°C, and (c) RSF treated at 100°C, showing the crystalline and amorphous contributions to the overall diffraction profiles.

2.5. Dynamic Light Scattering Analysis

Table S5. Z-average hydrodynamic diameter and polydispersity index (PDI) of untreated and thermally modified regenerated silk fibroin (RSF) obtained from three independent DLS measurements.

Sample	1	2	3

**RSF**	Z-average: 131.8 nm<br>PDI: 0.294	Z-average: 160.7 nm<br>PDI: 0.288	Z-average: 161.6 nm<br>PDI: 0.264
**RSF at 50°C**	Z-average: 243.7 nm<br>PDI: 0.398	Z-average: 236.3 nm<br>PDI: 0.379	Z-average: 208.4 nm<br>PDI: 0.355
**RSF at 100°C**	Z-average: 1062 nm<br>PDI: 0.547	Z-average: 893.0 nm<br>PDI: 0.446	Z-average: 1001 nm<br>PDI: 0.414

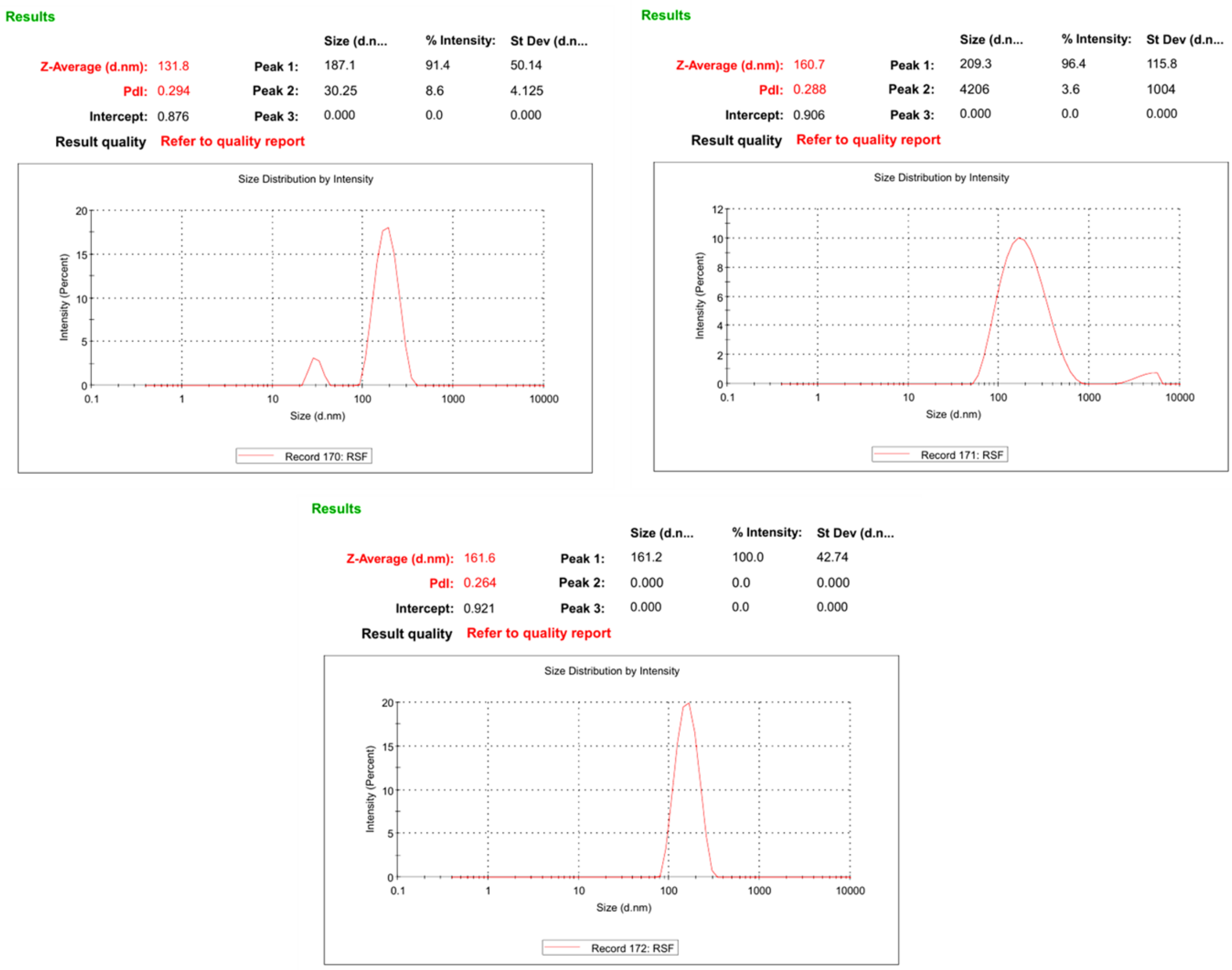

Figure S12. DLS size distribution and Z-average/PDI values of untreated RSF from three independent measurements.

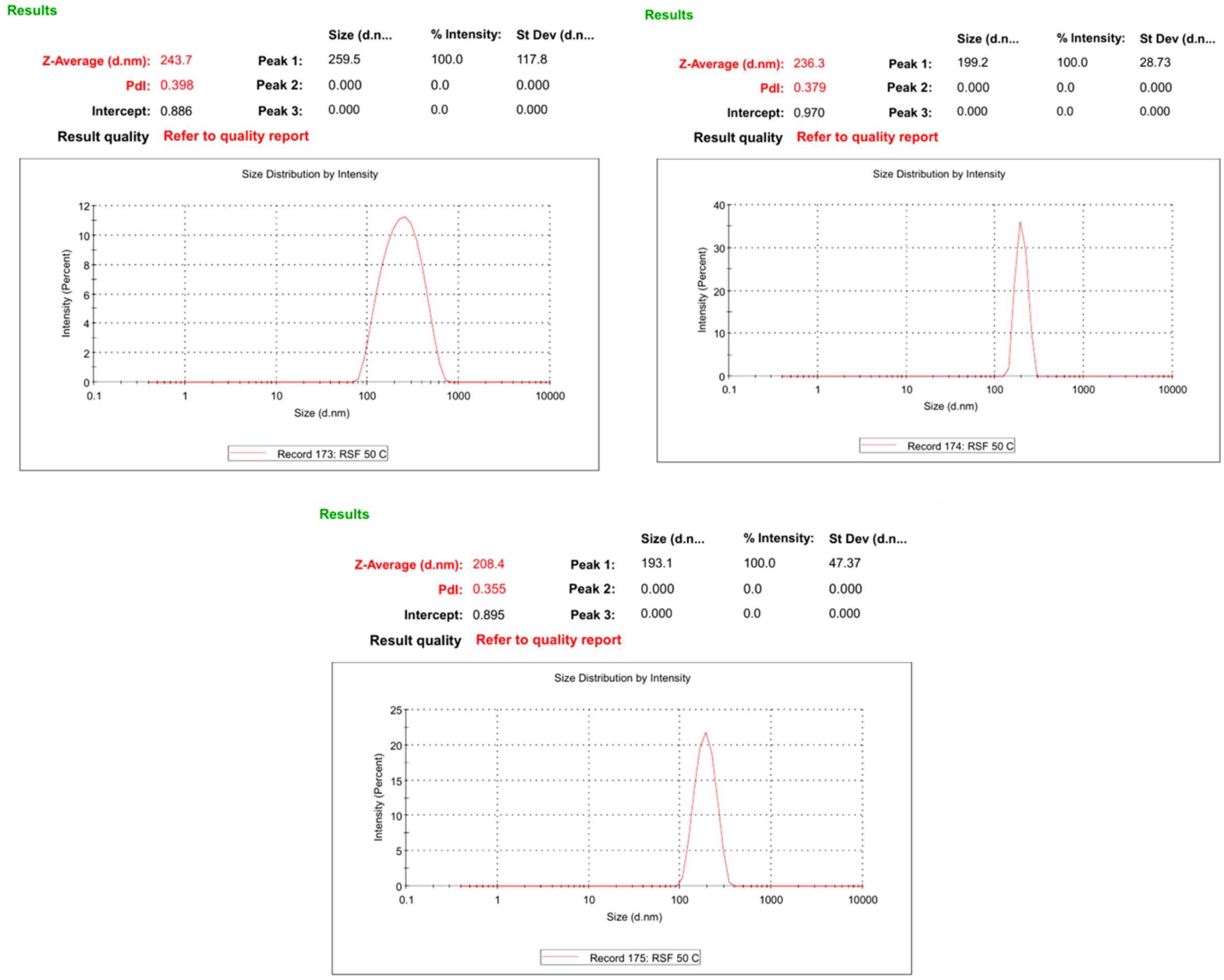

Figure S13. DLS size distribution and Z-average/PDI values of RSF treated at 50 °C from three independent measurements.

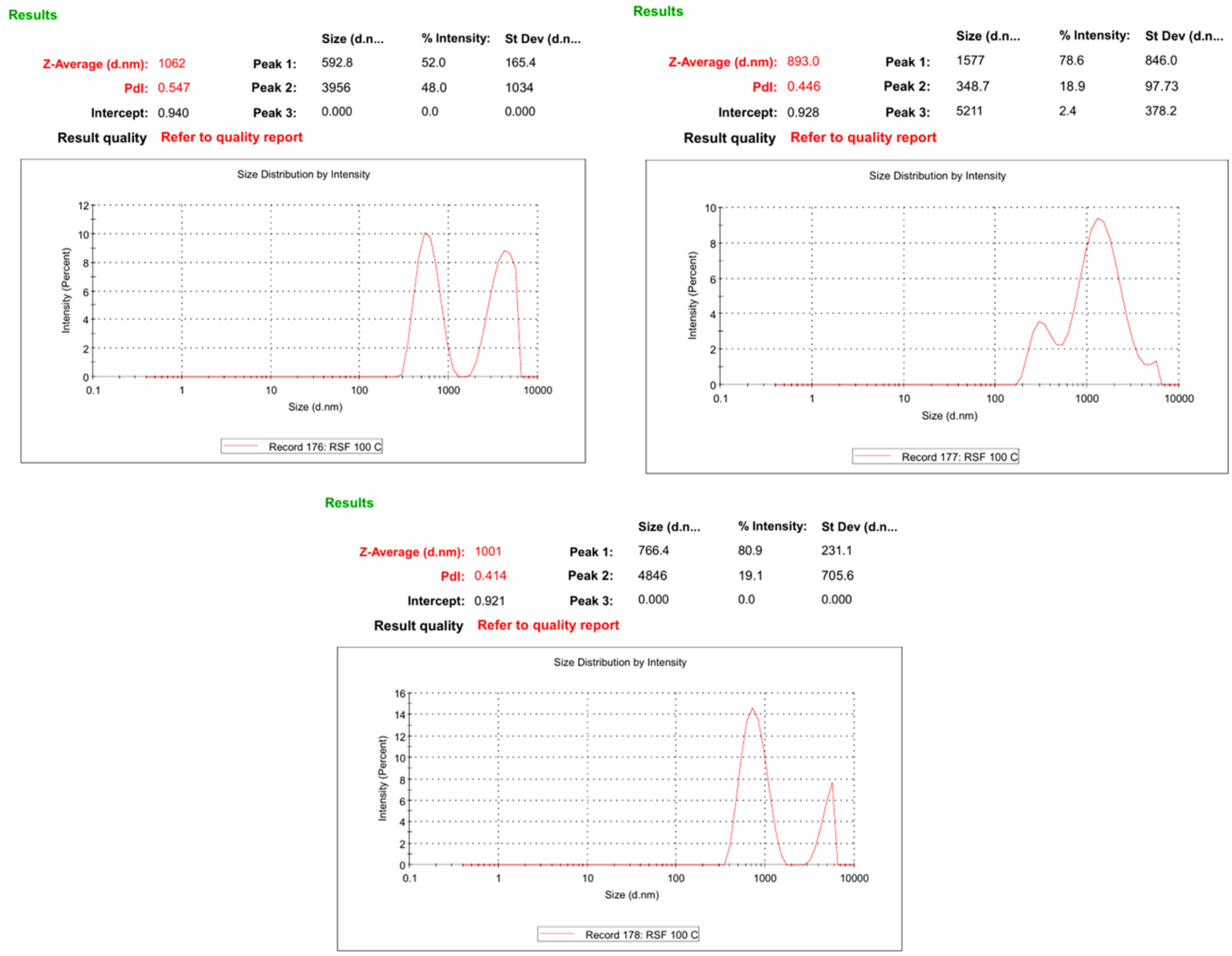

Figure S14. DLS size distribution and Z-average/PDI values of RSF treated at 100 °C from three independent measurements.

2.6. Atomic Force Microscopy

Table S6. Roughness measurements of untreated RSF and thermal-modified RSF at 50°C and 100°C.

SI.No	Samples	Average Roughness Ra	RMS Roughness Rq
1	RSF	16.40 nm	23.27 nm
2	RSF at 50°C	16.43 nm	24.61 nm
3	RSF at 100°C	19.48 nm	27.32 nm

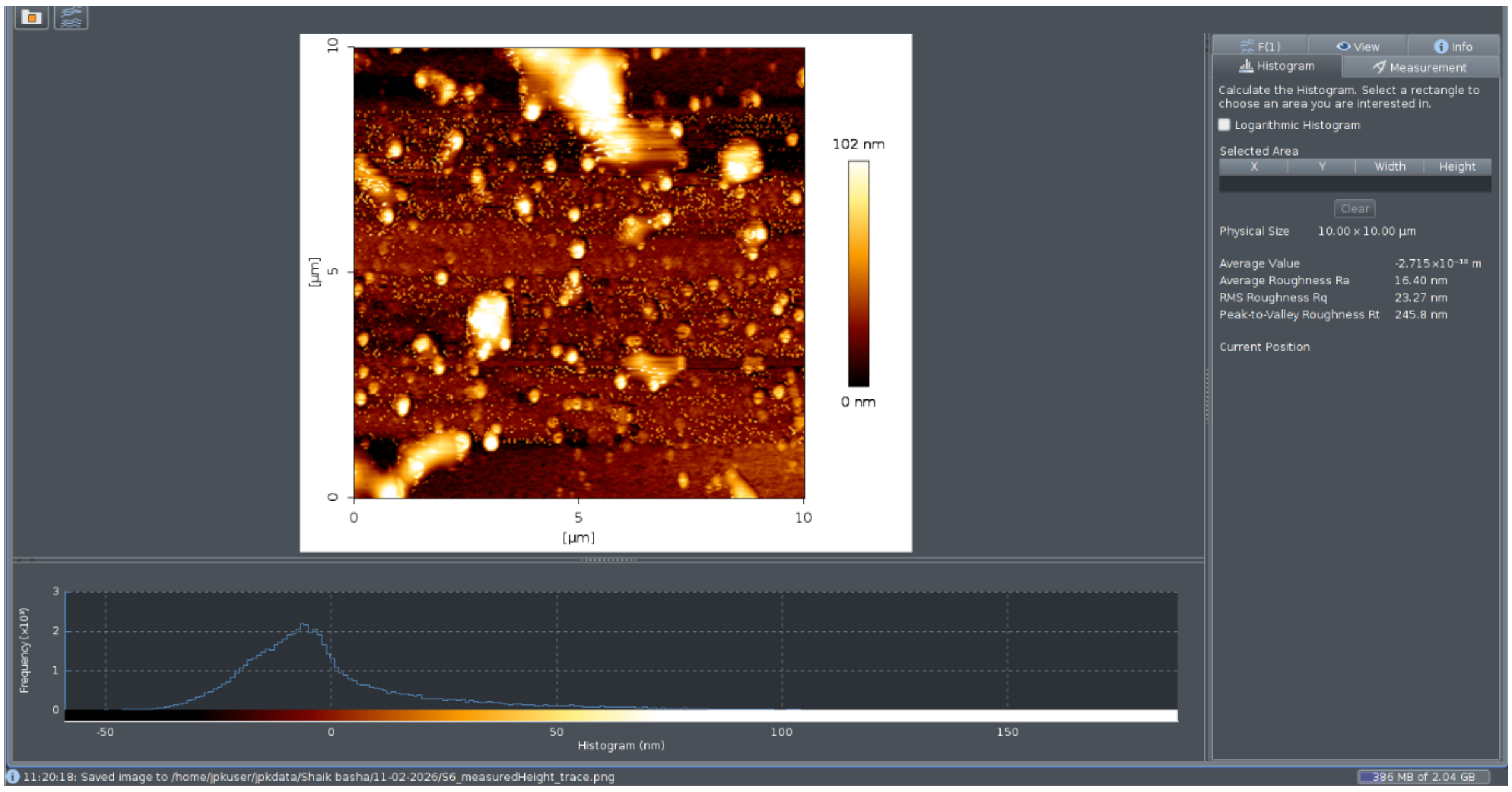

Figure S15. The roughness measurements of RSF.

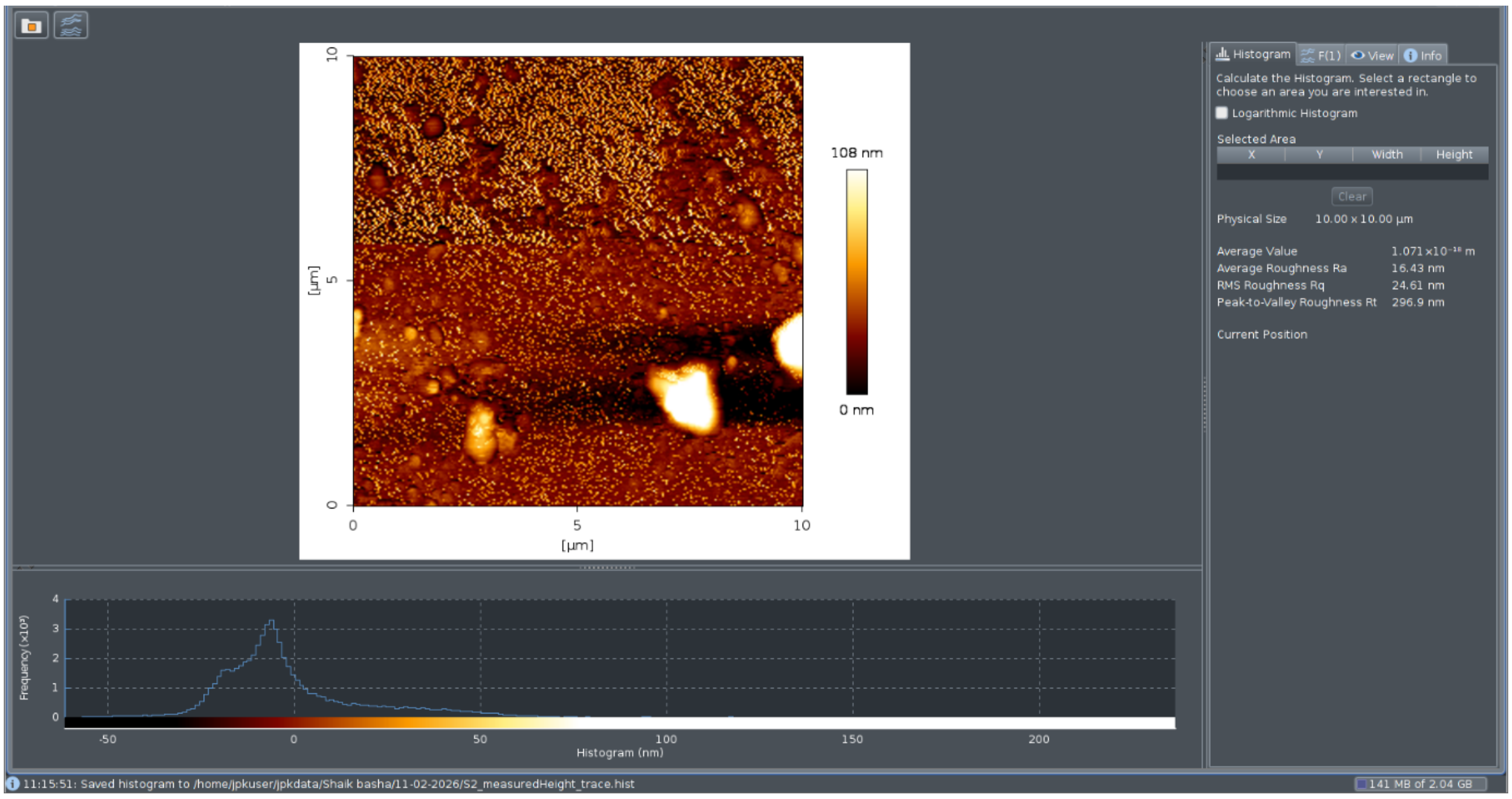

Figure S16. The roughness measurements of RSF at 50°C.

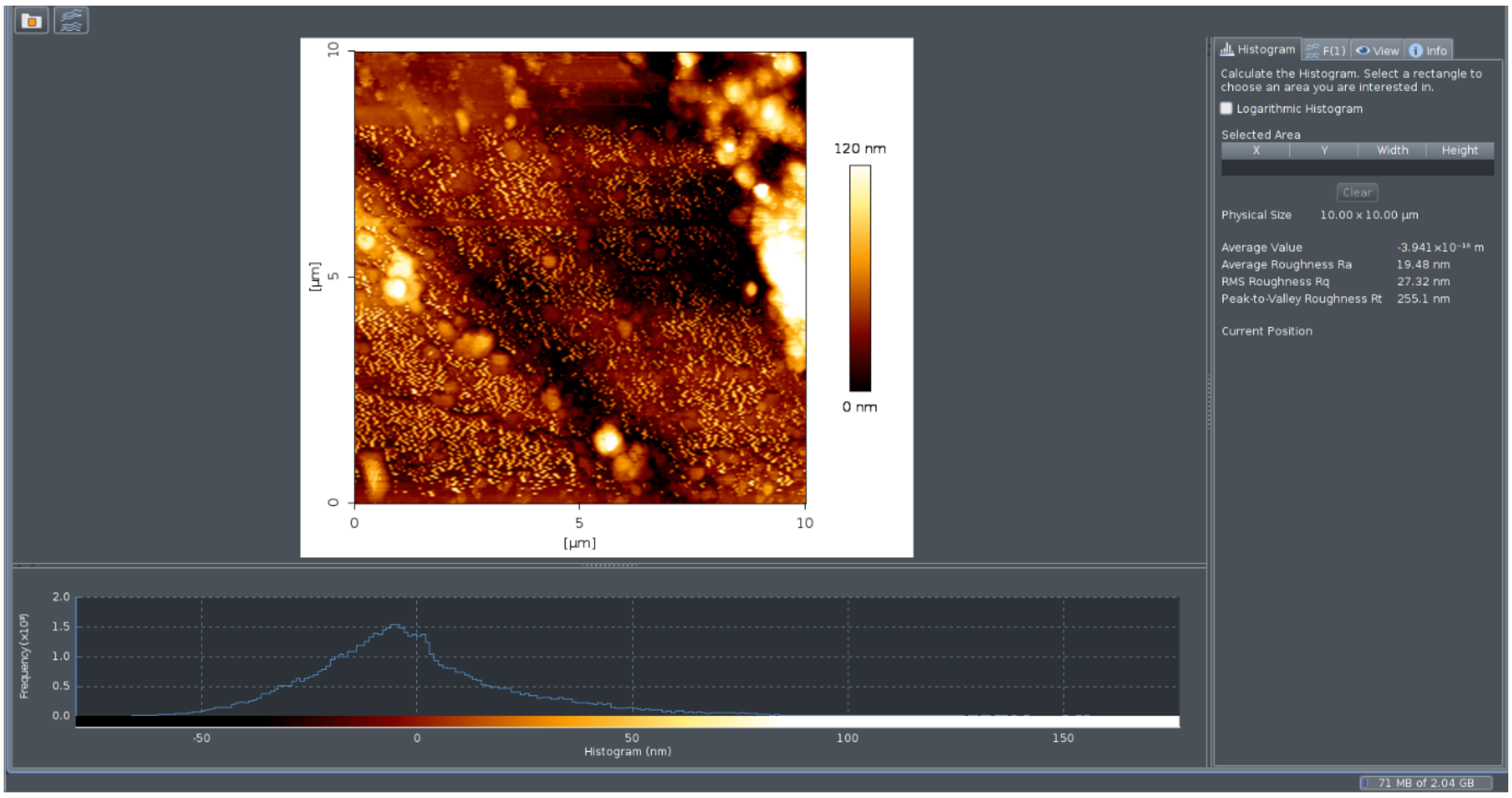

Figure S17. The roughness measurements of RSF at 100°C.

References

1. Basha S, Mukunda DC, Chandra S, et al. Label-free evaluation of protein aggregation by a point-of-care fluorometric device. *Int J Biol Macromol*. Published online January 2026:150333. doi:10.1016/j.ijbiomac.2026.150333

2. Puerta M, Peresin MS, Restrepo-Osorio A. Effects of Chemical Post-treatments on Structural and Physicochemical Properties of Silk Fibroin Films Obtained From Silk Fibrous Waste. *Front Bioeng Biotechnol*. 2020;8. doi:10.3389/fbioe.2020.523949
